# Ferroelectricity in Dipolar Liquids: The Role of Annealed Positional Disorder

**DOI:** 10.1021/acs.jpcb.5c08120

**Published:** 2026-06-29

**Authors:** Maria Grazia Izzo

**Affiliations:** Department of Molecular Sciences and Nanosystems, 19047 Ca’ Foscari University of Venice, Via Torino 155, Venezia Mestre 30172, Italy

## Abstract

Ferroelectric ordering in polar liquids has been observed in numerical simulations and liquid-crystal experiments. Within the mean-field framework, this behavior remains associated with the sample-shape-dependent surface contribution to the free energy, which does not vanish in the thermodynamic limit due to the long-range nature of dipolar interactions. Yet, numerical simulations performed under conducting periodic boundary conditions, for which the surface contribution vanishes, still exhibit ferroelectric order, pointing to an intrinsic bulk origin of the transition. Moving beyond the mean-field approximation, Kirkwood’s seminal study of the dielectric properties of polar liquids emphasized the role of hindered dipolar rotation in shaping the corresponding pair correlations. In Kirkwood’s analysis, hindered rotation stems from the mean force between nearest-neighbor dipoles, placing the focus on local structure. Introducing a different perspective while retaining the central role of hindered rotation in the onset of ferroelectricity, the present study establishes, as an original finding, that annealed averaging of dipolar interactions over positional disorder generates hindered dipolar rotation that favors dipole alignment and can drive a bulk ferroelectric phase transition. As a result, unlike approaches centered on local structure, ferroelectricity emerges not in spite of the liquid nature, but because of it. Annealed averaging over positional disorder defines an effective dipolar interaction that is shorter-ranged than the bare potential. This is analogous to the Keesom interaction, where screening arises from annealed dipolar disorder. Derived within classical density functional theory, these findings are exact for dimensions *d* → ∞ and remain valid within the optimized cluster expansion for *d* ≥ 3.

## Graphical abstract

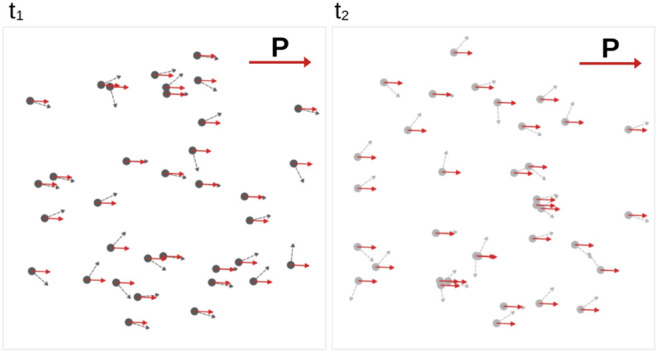

## 1 Introduction

The possibility of a ferroelectric phase transition in dipolar liquids traces back to the studies of Debye, Onsager, and Kirkwood.^1–3^ Following numerical simulations of dipolar liquids have reported transitions toward dipole-ordered states^4–9^ while recent experiments in liquid-crystals have provided evidence for a ferroelectric nematic phase.^10–12^ A renewed interest in the topic is further supported by recent findings showing that supercooled water in its low-density phase exhibits properties consistent with a ferroelectric phase.^13,14^ In Ref.^13^ it is furthermore shown how the liquid–liquid phase transition in supercooled water may be driven by a ferroelectric phase transition. A ferroelectric phase transition is characterized by the spontaneous emergence of a macroscopic polarization below a critical value of suitable thermodynamic control parameters, such as temperature or pressure. For liquids as well as solids, the order parameter of a ferroelectric phase transition is the macroscopic polarization vector per particle, **p̅**. In the case of a polar liquid of *N* particles labeled by *i* = 1, ···, *N*, each carrying a dipole moment *pd̂*_*i*_, it is defined as

1 $$\mathrm{p̅}=\left\langle \underset{N\to \infty }{\lim}\frac{1}{N}p\overset{N}{\underset{i=1}{\sum }}{\mathrm{d̂}}_{i}\right\rangle =\underset{T\to \infty }{\lim}\frac{1}{T}\overset{T}{\underset{\tau =0}{\sum }}\underset{N\to \infty }{\lim}\frac{1}{N}p\overset{N}{\underset{i=1}{\sum }}{\mathrm{d̂}}_{i}\left(\tau \right)$$

where *p* is the magnitude of the rigid particle dipole moment, ⟨⟩ denotes the ensemble average, τ is the time variable, and ergodicity allows one to set the equality in eq 1. In the paraelectric phase **p̅** = 0, whereas in the ferroelectric phase **p** ≠ 0, corresponding to the spontaneous breaking of continuous rotational symmetry. In a solid, where dipoles occupy fixed lattice sites, microscopic configurations corresponding to a ferroelectric phase are readily identified. The dipoles align so that the lattice-averaged polarization remains nonzero and essentially configuration independent, yielding a finite ensemble average. In a liquid, an analogous picture emerges once the lattice constraint is removed and individual dipoles are free to move in space. A ferroelectric liquid phase is then characterized by a nonzero single-particle dipole moment averaged over all particles, whose value remains approximately configuration independent, as in the solid. A schematic illustration is shown in Figure 1. However, while solids possess a frozen lattice, so that only dipolar dynamics must be constrained to maintain ferroelectric order, in liquids the emergence of more complex ferroelectric ordering, such as chiral order, can also constrain translational motion in addition to dipolar rotations. A nonzero macroscopic polarization alone does not establish a ferroelectric phase transition. In the thermodynamic limit, the latter is characterized by nonanalytic behavior of the free energy or its derivatives with respect to a control parameter, as prescribed by the Ehrenfest classification.^15^ If a phase transition is intrinsic to the bulk, its existence and critical behavior must be independent of boundary conditions, surface terms, and sample shape. This becomes especially relevant for long-range interactions, such as dipolar potential, as discussed below.

**Figure 1:**
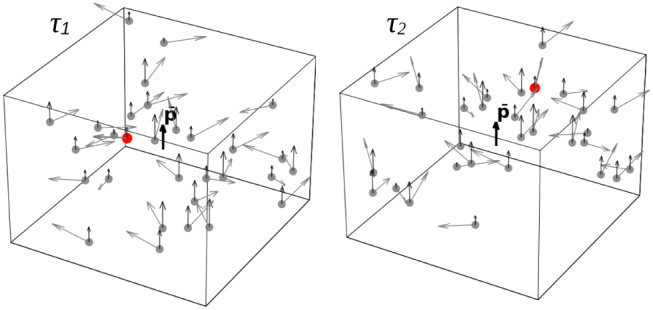
Schematic representation of two configurations of a dipolar liquid in three dimensions at times τ_1(2)_ in the ferroelectric phase. Gray arrows show the orientations of the particle dipole moments, thin black lines represent their projections along the direction of the macroscopic polarization per particle, shown by the thick black arrow at the center of the simulation box. Simple ferroelectric order, set only by a macroscopic polarization, does not constrain translational degrees of freedom and the particle highlighted in red is free to move in space, independently of the orientation of the dipole that it carries.

Classical density functional theory (DFT) provides a formally exact framework in which the free energy, *F*, of a many-body system is expressed as a functional of the one-body density field, ρ̃.^16^ The equilibrium function ρ̃ can be obtained by applying a variational principle to *F*[ρ̃]. Owing to its unbiased formulation, classical DFT is a powerful tool for the study of phase transitions. Moreover, it applies to both crystalline^17–19^ and liquid phases and, when combined with replica theory,^20^ can be further extended to glassy states.^21^ A common DFT scheme consists in decomposing the pair potential, *v*, into a reference potential, *v*_0_, the pair potential acting in the reference system, and a perturbative term, *w*_*p*_.^16^ In the case of dipolar liquids, *v*_0_ is the hard-sphere or the Lennard-Jones potential, and *w*_*p*_ is the dipolar interaction. The family of intermediate potentials

2 $$v_{\lambda }\left(r,{\mathrm{r̂}}_{ij},{\mathrm{d̂}}_{i},{\mathrm{d̂}}_{j}\right)=v_{0}\left(r,{\mathrm{r̂}}_{ij}\right)+\lambda w_{p}\left(r,{\mathrm{r̂}}_{ij},{\mathrm{d̂}}_{i},{\mathrm{d̂}}_{j}\right),,0\leq \lambda \leq 1$$

is then introduced to parametrize an adiabatic path from the reference system (λ = 0) to the fully interacting system (λ = 1).^16^ In eq 2, *d̂* is the dipole unit vector, subscripts *i* and *j* indicate association with particle *i* or *j*, respectively, **r** is a space vector and $r_{ij}={r\mathrm{r̂}}_{ij}=r_{i}-r_{j}$ . The free energy functional, in its more general DFT formulation, is

3 $$F\left[\mathrm{\rho ̃}\right]=F_{v_{0}}\left[\mathrm{\rho ̃}\right]+F\left[\mathrm{\rho ̃}\right]$$

where $F_{v_{0}}\left[\mathrm{\rho ̃}\right]$ is the free energy of the reference system and F is the excess free energy associated with *w*_p_,^16^

$$F\left[\mathrm{\rho ̃}\right]=\frac{1}{2}\int_{0}^{1}d\lambda \int dr_{i}dr_{j}d{\mathrm{d̂}}_{i}d{\mathrm{d̂}}_{j}\mathrm{\rho ̃}\left(r_{i},{\mathrm{d̂}}_{i}\right)g_{\lambda }^{\left(2\right)}\left(r_{ij},{\mathrm{d̂}}_{i},{\mathrm{d̂}}_{j}\right)\mathrm{\rho ̃}\left(r_{j},{\mathrm{d̂}}_{j}\right)w_{p}\left(r_{ij},{\mathrm{d̂}}_{i},{\mathrm{d̂}}_{j}\right)$$ 4

with d*d̂* being the differential solid angle element. In eq 4, $g_{\lambda }^{\left(2\right)}\left(r_{ij},{\mathrm{d̂}}_{i},{\mathrm{d̂}}_{j}\right)$ is the pair correlation function of the full system governed by the potential *v*_*λ*_(*r*, *r̂*_*ij*_, *d̂*_*i*_, *d̂*_*j*_) in eq 2. The one-particle density field for dipolar liquids is

5 $$\mathrm{\rho ̃}\left(r,\mathrm{d̂}\right)=\overset{N}{\underset{i=1}{\sum }}\delta \left(\mathrm{d̂}-{\mathrm{d̂}}_{i}\right)\delta \left(r-r_{i}\right)=\rho \left(r\right)\zeta \left(r,\mathrm{d̂}\right)$$

where ρ­(**r**) and ζ­(**r**, *d̂*) are respectively the particle number density marginalized over dipole orientation and the probability distribution of dipole orientation at **r**. δ­() is the Dirac delta function. As appropriate for a liquid, spatial homogeneity is assumed, making ρ̃(**r**, *d̂*) independent of **r**. However, no assumption is imposed on the dipole orientational probability distribution, thereby allowing for possible dipolar ordering within the liquid. ρ̃ then factorizes as

6 ρ̃=ρζ(d̂)

where ρ is the particle number density. In a DFT scheme the physical insight and technical challenges are entirely encoded in the characterization of $g_{\lambda }^{\left(2\right)}$ . The lowest level of approximation is a mean-field theory, where $g_{\lambda }^{\left(2\right)}=1$ . The excess free-energy functional for the one-particle density in eq 6 under this approximation becomes

7 $$F^{\mathrm{MF}}\left[\mathrm{\rho ̃}\right]=\frac{1}{2}N\rho \int d{\mathrm{r̂}}_{ij}r^{2}drd{\mathrm{d̂}}_{i}d{\mathrm{d̂}}_{j}w_{p}\left(r,{\mathrm{r̂}}_{ij},{\mathrm{d̂}}_{i},{\mathrm{d̂}}_{j}\right)$$

If one considers the bare dipolar interaction potential, e.g., in three-dimensional space,

$$w_{p}^{\left(3\right)}\left(r,{\mathrm{r̂}}_{ij},{\mathrm{d̂}}_{i},{\mathrm{d̂}}_{j}\right)=-p^{2}\frac{1}{r^{3}}\left[3\left({\mathrm{d̂}}_{i}\cdot {\mathrm{r̂}}_{ij}\right)\left({\mathrm{d̂}}_{j}\cdot {\mathrm{r̂}}_{ij}\right)-{\mathrm{d̂}}_{i}\cdot {\mathrm{d̂}}_{j}\right]$$ 8

the associated integral in eq 7 exhibits conditional convergence.^4,22–25^ Its value depends on the order of integration over the variables, *r* and r̂_*ij*_, both in the limits of short- and long-range interparticle separations. To avoid conditional convergence at short *r*, a physically motivated way is to take *w*_*p*_ vanishes inside the core region of *v*_0_, where it is indeed ineffective due to the hard-core repulsion. Alternatively, allowing for an arbitrary expression of *w*_*p*_ within the core region, the spatial integral of the dipolar interaction may be evaluated over the region outside a spherical cavity, with the cavity radius then taken to zero.^23^ However, it has been shown that the resulting free energy depends on the arbitrary definition of the perturbing potential within the core region.^26^ This approach therefore will not be considered further. Both the Debye and Onsager models, as well as the Wertheim mean-spherical approximation,^27^ can be retrieved within this mean-field scheme, each corresponding to a specific assignment of the perturbing potential inside the core.^26^ Due to the conditional convergence at long interparticle distances, the mean-field contribution to the excess free energy depends on the macroscopic shape of the sample.^28^ An intuitive picture emerges by considering an ellipsoidal sample with semiaxes α*R*_*c*_, β*R*_*c*_, and γ*R*_*c*_ along *x̂*, *ŷ*, and *ẑ*, respectively (Figure 2), and approximating the surface term in eq 7 associated with the ellipsoidal geometry, $F_{S_{e}}^{\mathrm{MF}}$ , by octahedral quadrature.^29^ The octahedral quadrature approximates angular integrals over three-dimensional space by a discrete sum over the six Cartesian directions, each with weight 2π/3. Within this approximation,

$$F_{S_{e}}^{\mathrm{MF}}\left(R_{c}\right)=\frac{1}{2}N\rho \frac{2\pi }{3}\underset{{\mathrm{r̂}}_{k}=\pm \mathrm{x̂},\pm ŷ,\pm ẑ}{\sum }w_{p}^{\left(3\right)}\left(1,\frac{{\mathrm{r̂}}_{k}}{{\mathrm{d̂}}_{i},{\mathrm{d̂}}_{i}}\right)\ln\left(R\left({\mathrm{r̂}}_{k}\right)\right)$$ 9

where *x̂*, *ŷ*, *ẑ* are the Cartesian directions and *R*(*r̂*_*k*_) is the distance from the center to the surface of the ellipsoid along direction *r̂*_*k*_, i.e., *R*(±*x̂*) = α*R*_*c*_, *R*(±*ŷ*) = β*R*_*c*_, and *R*(±*ẑ*) = γ*R*_*c*_. Taking *d̂*_*i*_ = *x̂*, in the macroscopic limit *R*_*c*_ → ∞, one obtains

10 $$F_{S_{e}}^{\mathrm{MF}}=-\frac{1}{2}N\rho p^{2}\frac{2\pi }{3}{\mathrm{d̂}}_{i}\cdot {\mathrm{d̂}}_{j}\left[4\ln\left(\alpha \right)-2\ln\left(\beta \right)-2\ln\left(\gamma \right)\right]$$

The surface term is thus different from zero and shape dependent. If the quantity in the brackets is positive, the bulk system exhibits a tendency toward dipole alignment, if negative it favors antiparallel dipole alignment. When the ellipsoid reduces to a sphere, α = β = γ, and $F_{S_{e}}^{\mathrm{MF}}$ vanishes. For a bulk homogeneous and thus isotropic system, the bulk contribution to the integral in eq 7 vanishes. Indeed, isotropy translates in a uniform distribution of *r̂*_*ij*_ over the solid angle, so that integration over *r̂*_*ij*_ reduces to an average over the solid angle. In particular, $\left\langle \left({\mathrm{d̂}}_{i}\cdot {\mathrm{r̂}}_{ij}\right)\left({\mathrm{d̂}}_{j}\cdot {\mathrm{r̂}}_{ij}\right)\right\rangle =\frac{1}{d}{\mathrm{d̂}}_{i}\cdot {\mathrm{d̂}}_{j}$ , where *d* is the spatial dimension. This follows directly from rotational invariance, which implies $\left\langle {\mathrm{r̂}}_{ij}^{\alpha }{\mathrm{r̂}}_{ij}^{\beta }\right\rangle =\frac{1}{d}\delta_{\alpha \beta }$ , where α and β denote the components of the unit vector *r̂*_*ij*_. This offsets the remaining term of the dipolar potential, thus making the bulk contribution to the integral in eq 7 vanish. For a homogeneous system with one-particle density given in eq 6, $F^{\mathrm{MF}}$ thus reduces to a pure surface contribution. Note that the conditional convergence of real-space integrals of the dipolar potential persists even in the limit *d* → ∞. Most theoretical studies predicting the occurrence of a ferroelectric phase transition in dipolar liquids rely on mean-field approximation, or assume the mean-field contribution as the leading term in the free energy.^23,24,28,30^ The resulting ferroelectric phase transition cannot thus be regarded as a genuine bulk phase transition. The first question addressed in this study is the following: is this conclusion merely a consequence of the mean-field approximation? Recast in more general terms:

**Figure 2:**
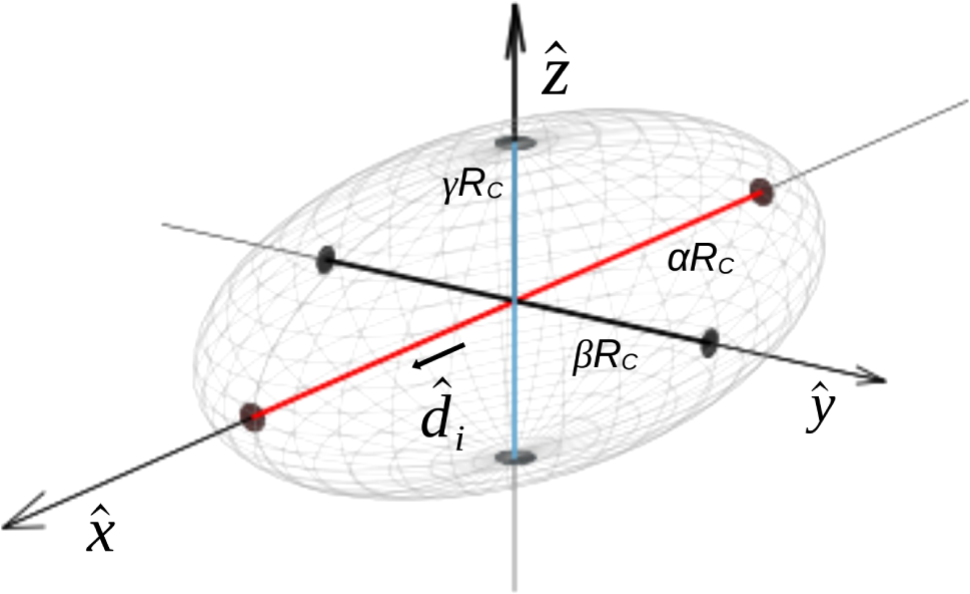
Sketch of the ellipsoidal sample used to estimate, via octahedral quadrature, the surface contribution to the excess free energy associated with the dipolar potential in a spatially homogeneous system within the mean-field approximation.

(i) Does a ferroelectric phase transition exist in dipolar liquids as an intrinsic bulk property?

This issue is far from being only conceptual. Consider, for instance, water. The proposed liquid–liquid phase transition in the supercooled regime has been invoked to explain the thermodynamic anomalies of bulk water. Ref 13 showed that, insofar as water can be described as a dipolar liquid, such a transition may be driven by ferroelectric ordering. Since both the liquid–liquid phase transition and the thermodynamic anomalies are intrinsic bulk properties, consistency requires such to be the underlying ferroelectric phase transition. Were this not the case, one would need to investigate the role of interactions other than dipolar in accounting for the ferroelectric order observed in the low-density phase of supercooled water in numerical simulations.^13,14^ Such interactions may nonetheless influence nonuniversal features of the transition. However, if dipolar interactions are not the primary driving mechanism, the criteria for identifying such alternative interactions and their impact would be different. The surface contribution to the extra free energy in eq 7 vanishes when the liquid is embedded in a conducting medium, as well as in numerical simulations under Ewald summation with conducting periodic boundary conditions. Yet numerical simulations consistently report ferroelectric ordering in dipolar liquids under these conditions,^4–6,8^ including those analyzed in Ref.^13^, providing support for a positive answer to the questions raised above. However, since identifying a genuine phase transition in finite-size simulations remains nontrivial, a theoretical understanding is desirable.

In dipolar liquids in the absence of ferroelectric order, free rotational motion of the dipoles screens dipolar interactions^31,32^ rendering the bare long-range dipolar potential effectively shorter ranged leading to the so-called Keesom interaction.^33^ Conversely, whether the particles translational motion can induce a screening of the dipolar interaction has been overlooked. In a liquid, particle positions are not quenched but fully equilibrated and treated as statistical variables in the equilibrium ensemble. In the following, this property will be referred to as annealed positional disorder. As a consequence, the free energy, up to the ideal-gas contribution, is expressed in terms of the partition function *Z* as

11 $$F=-\frac{1}{\beta }\ln Z=-\frac{1}{\beta }\ln\langle e^{-\beta \sum_{i<j}[v_{0}(r_{ij})+w_{p}(r_{ij},{\mathrm{r̂}}_{ij},{\mathrm{d̂}}_{i},{\mathrm{d̂}}_{j})]}\rangle_{\{r_{i},{\mathrm{d̂}}_{i}\}}$$

where $\beta =\frac{1}{k_{B}T}$ , *T* is the temperature and *k*_B_ the Boltzmann constant. The explicit dependence of *r* on the particle indices has been introduced for clarity, the case λ = 1 of the family of potentials in eq 2 is considered, and $\langle \rangle_{\{r_{i},{\mathrm{d̂}}_{i}\}}$ denotes the equilibrium ensemble average over all configurations of particle positions **r**_*i*_ and dipole orientations *d̂*_*i*_. Since the system is assumed to remain liquid in its positional degrees of freedom even upon the onset of dipolar order, the distribution of *r̂*_*ij*_ is always isotropic within the present framework. The screening of the dipolar interaction by translational degrees of freedom can then be understood as arising from an annealed average over *r̂*_*ij*_. Given a two-body potential interaction *W*(*r*_*ij*_, *r̂*_*ij*_, *d̂*_*i*_, *d̂*_*j*_), its corresponding annealed average over *r̂*_*ij*_, *W̅*(*r*_*ij*_, *d̂*_*i*_, *d̂*_*j*_) is defined through $e^{-\beta \mathrm{W̅}(r_{ij},{\mathrm{d̂}}_{i},{\mathrm{d̂}}_{j})}=\langle e^{-\beta W(r_{ij},{\mathrm{r̂}}_{ij},{\mathrm{d̂}}_{i},{\mathrm{d̂}}_{j})}\rangle_{{\mathrm{r̂}}_{ij}}$ , where $\langle \rangle_{{\mathrm{r̂}}_{ij}}$ denotes the ensemble average over the possible configurations of *r̂*_*ij*_, which, in the case of a liquid, coincides with an average over a uniform distribution on the solid angle. The bare potential *W*(*r*_*ij*_, *r̂*_*ij*_, *d̂*_*i*_, *d̂*_*j*_) can be the dipolar interaction itself or a renormalization thereof that effectively accounts for many-body interactions. In either case, the screened dipolar interaction *W̅* is well-defined, only if the associated free energy

12 $$\mathrm{F̅}=-\frac{1}{\beta }\ln Z=-\frac{1}{\beta }\ln\langle e^{-\beta \sum_{i<j}[v_{0}(r_{ij})+\mathrm{W̅}(r_{ij},{\mathrm{d̂}}_{i},{\mathrm{d̂}}_{j})]}\rangle_{\{r_{i},{\mathrm{d̂}}_{i}\}}$$

coincides, up to an irrelevant additive constant, with the free energy in eq 11. The second issue raised in this study is the following:

(ii) Can the annealed positional disorder in the liquid screen the dipolar potential? More specifically, can annealed averaging over *r̂*_*ij*_ generate an isotropic, short-ranged dipolar interaction that fully characterizes the free energy of the liquid?

It is worth emphasizing that, in order to answer these questions, for a liquid, where the one-particle density is independent of **r**, the free energy cannot be computed within the mean-field approximation, since within this framework the only nonvanishing contribution is the surface term. The question becomes thus ill posed, since no isotropic potential in **r**-space can reproduce a purely shape-dependent contribution. A first indication that such an effective potential may exist, possibly inducing a ferroelectric phase transition, is provided by the observation that both dipolar and Heisenberg fluids with purely short-range spin–spin interactions exhibit orientationally ordered phases and display similar phase diagrams.^5,30,34–36^ A ferroelectric phase transition in a liquid implies dipolar ordering without crystallization, a condition expected to hold when the dipolar interaction is a sufficiently weak perturbation of an otherwise **r**-isotropic reference potential. The screened dipolar interaction introduced above can then be interpreted as arising from the screening of the perturbing dipolar potential by the **r**-isotropic reference potential, in analogy with screening mechanisms such as the random-phase approximation (RPA) for fluids,^16^ Debye–Hückel screening in ionic solutions,^16^ and optimized cluster expansion of the free energy.^16,37^

Addressing the questions raised above calls for a theoretical framework beyond the mean-field approximation. The role of dipolar pair correlations in shaping the dielectric properties of polar liquids was first recognized in the seminal study by Kirkwood.^3^ Following Ref 3, the dipolar pair correlation function is given by the following formally exact expression (in the absence of ferroelectric order):

13 $$\left\langle {\mathrm{d̂}}_{i}\cdot {\mathrm{d̂}}_{j}\right\rangle =\frac{\int d{\mathrm{d̂}}_{i}d{\mathrm{d̂}}_{j}dr_{ij}{\mathrm{d̂}}_{i}\cdot {\mathrm{d̂}}_{j}e^{-\beta V(r_{ij},{\mathrm{d̂}}_{i},{\mathrm{d̂}}_{j})}}{\int d{\mathrm{d̂}}_{i}{\mathrm{d̂}}_{j}dr_{ij}e^{-\beta V(r_{ij},{\mathrm{d̂}}_{i},{\mathrm{d̂}}_{j})}}$$

*V*(**r**_*ij*_, *d̂*_*i*_, *d̂*_*j*_) is the two-body potential of mean force between a pair of molecules with dipole orientations *d̂*_*i(j)*_ and center-of-mass distance **r**_*ij*_. It can be obtained by marginalizing the full configurational Boltzmann weight $e^{-\beta V_{N}(\{r_{k},{\mathrm{d̂}}_{k}\})}$ over all degrees of freedom other than *d̂*_*i(j)*_ and **r**_*ij*_. *V*_*N*_ is the total potential energy of the system in the configuration {**r**_*k*_, *d̂*_*k*_}. In the case of pairwise additive interactions described by a two-body potential *v*(**r**_*ij*_, *d̂*_*i*_, *d̂*_*j*_), $V_{N}=\underset{i<j}{\sum }v(r_{ij},{\mathrm{d̂}}_{i},{\mathrm{d̂}}_{j})$ . By its very definition, *V*(**r**_*ij*_, *d̂*_*i*_, *d̂*_*j*_) coincides, up to an additive constant, with $-\frac{1}{\beta }\log g^{\left(2\right)}\left(r_{ij},{\mathrm{d̂}}_{i},{\mathrm{d̂}}_{j}\right)$ , where *g* ^(2)^(**r**_*ij*_, *d̂*_*i*_, *d̂*_*j*_) is the pair correlation function appearing in eq 4 with λ = 1 if *v*(**r**_*ij*_, *d̂*_*i*_, *d̂*_*j*_) = *v*_0_(*r*_*ij*_) + *w*_*p*_(**r**_*ij*_, *d̂*_*i*_, *d̂*_*j*_). In schemes where many-body effects are reduced to effective pair interactions, the form *V*(**r**_*ij*_, *d̂*_*i*_, *d̂*_*j*_) = *V*_0_(*r*_*ij*_) + *W*(**r**_*ij*_, *d̂*_*i*_, *d̂*_*j*_) is typically retained, with *V*_0_ a reference potential and *W* a perturbative contribution. In full generality, eq 13 takes the form

14 $$\left\langle {\mathrm{d̂}}_{i}\cdot {\mathrm{d̂}}_{j}\right\rangle =\frac{\int d{\mathrm{d̂}}_{i}d{\mathrm{d̂}}_{j}dr_{ij}{\mathrm{d̂}}_{i}\cdot {\mathrm{d̂}}_{j}\mathrm{\rho ̃}\left(r_{i},{\mathrm{d̂}}_{i}\right)e^{-\beta V(r_{ij},{\mathrm{d̂}}_{i},{\mathrm{d̂}}_{j})}\mathrm{\rho ̃}\left(r_{j},{\mathrm{d̂}}_{j}\right)}{\int d{\mathrm{d̂}}_{i}{\mathrm{d̂}}_{j}dr_{ij}\mathrm{\rho ̃}\left(r_{i},{\mathrm{d̂}}_{i}\right)e^{-\beta V(r,{\mathrm{r̂}}_{ij},{\mathrm{d̂}}_{i},{\mathrm{d̂}}_{j})}\mathrm{\rho ̃}\left(r_{j},{\mathrm{d̂}}_{j}\right)}$$

The relevance of this latter quantity to the macroscopic polarization becomes apparent upon considering the square of **p̅** in eq 1. From eqs 13 and 14 the central role of the effective potential *V* clearly emerges: it sets the hindered rotation of the dipoles, which is directly encoded in the pair correlation function ⟨*d̂*_*i*_·*d̂*_*j*_⟩.^3^ A positive value of this latter quantity signals a tendency for dipole pairs to align. This can be readily illustrated by considering a Heisenberg fluid, i.e., a system of particles carrying vector spins, which can be identified with *d̂*_*i(j)*_ in eq 13, interacting via a short-ranged, isotropic ferromagnetic exchange potential, *V*(**r**_*ij*_, *d̂*_*i*_, *d̂*_*j*_) = *J*(*r*_*ij*_)*d̂*_*i*_·*d̂*_*j*_, with *J*(*r_ij_*) > 0. In this case, ⟨*d̂*_*i*_·*d̂*_*j*_⟩ > 0 as a direct consequence of the fact that *V* is minimized by parallel orientations of *d̂*_*i*_ and *d̂*_*j*_, a configuration that therefore has a larger weight than oppositely aligned orientations in the integral in eq 13. Any effective dipolar potential that is minimized when the interacting dipoles are parallel, and therefore favors dipole alignment, may be termed ferroelectric-like. The observations above suggest that the behavior of ⟨*d̂*_*i*_·*d̂*_*j*_⟩ in the paraelectric phase provides a criterion to assess the possibility of a ferroelectric phase transition. An explicit evaluation of *W* is nontrivial. In Ref. 3 it is approximate by the potential of average torque, *W*_0_, acting between a pair of nearest-neighbor dipoles. Restricting the description to nearest neighbors, a notion of local structure is implicitly introduced. Consistently, in Ref. 3 ⟨*d̂*_*i*_·*d̂*_*j*_⟩ depends on the average coordination number. When addressing the case of water, therefore, the focus is on tetrahedral coordination. No explicit microscopic characterization of *W*_0_ is provided, and the origin of hindered rotation is ascribed to a combination of dipole–dipole electrostatic interactions and other intermolecular forces, whose relevance depends on the specific system. This approach cannot account for differences in the behavior of liquid and solid phases with similar local structure. In the case of water, for example, it cannot account for why the low-density liquid phase may exhibit ferroelectric order, whereas hexagonal ice, despite having a closely related local structure, does not. What distinguishes a liquid from a solid is exactly the presence, in the former, of annealed positional disorder. This brings us to the third, and central, question of this manuscript:

(iii) Can the screened dipolar potential *W̅*, obtained from the annealed average over *r̂*_*ij*_ of the dipolar contribution *W* to the effective two-body interaction, induce hindered dipole rotation leading to a ferroelectric phase transition in the liquid?

If so, this would be a significant result: the driving mechanism for a bulk ferroelectric transition in dipolar liquids would then arise directly from the liquid character of the positional degrees of freedom, without invoking specific local structures or additional short-range interactions. It is worth emphasizing that the annealed averaging amounts to a marginalization of eq 13 over *r̂*_*ij*_, and therefore does not represent a local property. This outcome would point to a distinct perspective on the ferroelectric phase transition in dipolar liquids, with potentially far-reaching implications. For example, provided that water can be modeled as a dipolar liquid, the occurrence of a ferroelectric phase transition accompanying the high-to-low-density liquid phase transition in supercooled water^13^ could be directly traced back to the liquid nature of the system itself, with the driving mechanism for ferroelectric order already present in the paraelectric high-density phase. By contrast, within a Kirkwood-like framework in which local structure governs hindered rotation, ferroelectric order could arise as a consequence of the specific local ordering in the low-density phase. In this view, ferroelectricity would follow the high-to-low-density phase transition rather than drive it. A second consequence is that frustration of dipolar orientations associated with specific lattice arrangements in solids, such as in certain ice phases, would be absent in the liquid, characterized by annealed positional disorder.

The rest of the paper is organized as follows. In Section 2, a dipolar liquid within a generalized Onsager reaction-field framework^2^ is introduced, in which the shape-dependent surface contribution to the free energy vanishes. This enables an assessment of whether a genuine ferroelectric phase transition can occur, as posed in question (i). As noted above, the marginalization of the full configurational Boltzmann weight $e^{-\beta V_{N}(\{r_{k},{\mathrm{d̂}}_{k}\})}$ to derive *V*(**r**_*ij*_, *d̂*_*i*_, *d̂*_*j*_), or equivalently *g* ^(2)^(**r**_*ij*_, *d̂*_*i*_, *d̂*_*j*_), and then *W*(**r**_*ij*_, *d̂*_*i*_, *d̂*_*j*_), is a nontrivial task. A particularly transparent limit is obtained when many-body correlations are negligible, as in the case where the virial expansion can be truncated at second order. In this case, *V*(**r**_*ij*_, *d̂*_*i*_, *d̂*_*j*_) = *v*(**r**_*ij*_, *d̂*_*i*_, *d̂*_*j*_) and *W*(**r**_*ij*_, *d̂*_*i*_, *d̂*_*j*_) = *w*_*p*_(**r**_*ij*_, *d̂*_*i*_, *d̂*_*j*_). Since this truncation becomes exact in the limit *d* → ∞, under suitable conditions on the thermodynamic state of the system and interaction parameters, it is natural to examine this case first. This is addressed in Section 3. The virial expansion of dipolar liquids in the limit *d* → ∞, its reformulation within DFT, the conditions under which the truncation of the virial series becomes exact, and the scaling behavior of the free energy in this limit are discussed in Section 3.1. In the limit *d* → ∞, the link between annealed averaging over *r̂*_*ij*_ and the emergence of ferroelectricity in dipolar liquids becomes particularly clear, as discussed in Section 3.2. Issues (i)–(iii) can be addressed exactly in the limit *d* → ∞, as shown in Section 3.3. The analysis of the character of the screened dipolar interactoin, resulting from the annealed average of the bare dipolar potential over *r̂*_*ij*_, its interaction range, and its role in hindering dipole rotation and shaping both dipolar and positional pair correlation functions, is presented in Section 3.4. The generalization of the results obtained in the limit *d* → ∞ to finite *d* is addressed in Section 4. In three-dimensional systems, truncation of the virial expansion at second order generally provides a poor approximation. It is therefore desirable to derive a bare effective two-body potential interaction between dipoles incorporating many-body effects, analogous to *V*(**r**_*ij*_, *d̂*_*i*_, *d̂*_*j*_) in eq 13, from which an effective free-energy expression with a second-order virial truncation can be constructed. The annealed averaging can then be performed on *W*(**r**_*ij*_, *d̂*_*i*_, *d̂*_*j*_), rather than on the bare dipolar potential. Section 4.1 introduces the so-called optimized cluster expansion for classical fluids^16,37^ which provides an approximate expression for *W*. Section 4.2 analyzes the properties of its annealed average over *r̂*_*ij*_. Section 4.3 derives a simplified three-dimensional expression for the screened dipolar interaction annealed-averaged over *r̂*_*ij*_, whose mean-field free energy qualitatively reproduces the tendency toward ferroelectric ordering obtained using the optimized cluster expansion. Although widely applicable,^37^ the optimized cluster expansion remains an approximation. Results derived within this framework are therefore valid insofar as the underlying approximation provides a reliable description. A simpler route would be to generalize the *d* → ∞ results to finite *d* in systems where the virial expansion truncates at second order and correlations beyond pairwise are negligible. In the Supporting Information it is indeed shown that in this case the corresponding F is minimized by a ferroelectric state. However, the use of the optimized cluster expansion provides a broader scope for the present results, showing that they remain valid even when many-body correlations are incorporated into an effective pair potential. Conclusions are drawn in Section 5.

## 2 Dipolar Interaction: The Intrinsic Bulk Contribution

The existence of a genuine bulk ferroelectric phase transition in dipolar liquids can be assessed only under conditions where the shape-dependent surface term is suppressed. A possible route is to consider the reaction-field construction, originally introduced by Onsager.^2^ A generalization^4,25,38–40^ to more than one dipole inside the cavity is adopted here, following the approach of Kirkwood.^3^ A spherical cavity of radius *R*_*c*_ is carved out of the dipolar liquid, containing a certain number of dipoles. The liquid outside the cavity is modeled as a homogeneous and isotropic dielectric continuum with permittivity ϵ, and it is assumed to respond linearly to the polarization inside the cavity. This construction induces an additional two-body effective interaction between dipoles inside the cavity, encoding many-body effects due to the mean-field response of the liquid outside the cavity. The spherical geometry of the cavity enforces an isotropic response, free from contributions associated with macroscopic surface anisotropies. In the limit *R*_*c*_ → ∞, any macroscopic polarization must therefore originate from the bulk. The dipolar liquid is governed by the potential in eq 2, with λ = 1. The dipolar interaction inside the cavity is described by the following generalized expression, incorporating the Onsager construction:

15 $$w_{p}\left(r,{\mathrm{r̂}}_{ij},{\mathrm{d̂}}_{i},{\mathrm{d̂}}_{j};R_{c},\epsilon \right)=-p^{2}\left[{\left(\frac{l}{r}\right)}^{d}\left[d\left({\mathrm{d̂}}_{i}\cdot {\mathrm{r̂}}_{ij}\right)\left({\mathrm{d̂}}_{j}\cdot {\mathrm{r̂}}_{ij}\right)-{\mathrm{d̂}}_{i}\cdot {\mathrm{d̂}}_{j}\right]+\frac{f_{d}\left(\epsilon \right)}{R_{c}^{d}}{\mathrm{d̂}}_{i}\cdot {\mathrm{d̂}}_{j}\right]\theta \left(r-l\right)\theta \left(R_{c}-r\right)$$

where *f*_*d*_(ϵ) > 0 is a bounded function of ϵ, ∀ *d*. The last term in square brackets in eq 15 describes the contribution to the dipolar interaction arising from the liquid outside the cavity. θ­(*x*) is the Heaviside function and *l* is the hard-sphere diameter when *v*_0_ is a hard-sphere potential, or the effective interaction range when *v*_0_ is a Lenard-Jones potential. The factor θ­(*r* – *l*) ensures that the perturbative potential vanishes within the core region, as discussed in Section 1. The scaling factor *l* ^*d*^ sets the characteristic length scale associated with *w*_*p*_, *L* = *l*. To leading order in the limit *d* → ∞, *w*_*p*_(*r*) is nonzero only within a narrow region around *r* = *L,* ^20^ as can be readily inferred from its expression in eq 15. Hence, if *L* < *l*, *w*_*p*_ becomes ineffective as *d* → ∞. If *L* > l, the leading large-*d* behavior is the same as for *L* = *l*. Fixing *L* = *l* is then the more natural choice. The above construction assumes *R*_*c*_ > *l*. The effective pair potential in eq 15 satisfies both the stability and the temperedness conditions. Three remarks are in order regarding the contribution of the mean-field response of the liquid outside the cavity to the dipolar pair interaction inside the cavity in eq 15, $-p^{2}\frac{f_{d}\left(\epsilon \right)}{R_{c}^{d}}{\mathrm{d̂}}_{i}\cdot {\mathrm{d̂}}_{j}$ . (i) This term favors the alignment of two dipoles. This ferroelectric-like character, is a direct consequence of the assumption of a linear and isotropic response of the external medium, which determines the functional form of this contribution. (ii) In the liquid phase, ergodicity of the positional degrees of freedom implies an isotropic distribution of the intermolecular direction *r̂*_*ij*_ over the solid angle, as highlighted above. The annealed averaging over positional degrees of freedom is therefore performed accordingly. Assuming a spherical cavity, thereby modeling an isotropic response of the liquid outside the cavity, is thus coherent with the annealed averaging of the medium inside the cavity. One can anticipate that this averaging likewise imparts a ferroelectric-like character to the dipolar interaction. (iii) In the limit *d* → ∞ this contribution vanishes. This reflects the fact that many-body correlations disappear in this limit, as discussed in Section 3.

The bulk behavior is obtained in the limit *R*_*c*_ → ∞. Since $\langle w_{p}^{0}\rangle_{{\mathrm{r̂}}_{ij}}=0$ , as observed in Section 1, it is straightforward to verify that

16 $$\int r^{d-1}drd{\mathrm{r̂}}_{ij}w_{p}\left(r,{\mathrm{r̂}}_{ij},{\mathrm{d̂}}_{i},{\mathrm{d̂}}_{j};R_{c},\epsilon \right)=-p^{2}{\mathrm{d̂}}_{i}\cdot {\mathrm{d̂}}_{j}f_{d}\left(\epsilon \right)\frac{\Omega_{d}}{d}\left[1-{\left(\frac{l}{R_{c}}\right)}^{d}\right]$$

In the limit *d* → ∞, the right-hand side of eq 16 vanishes. For any function *g* of *w*_*p*_(*r*, *r̂*_*ij*_,*d̂*_*i*_, *d̂*_*j*_; *R*_*c*_, ϵ) admitting a Taylor expansion around *w*_*p*_ = 0, the following relation then holds:

17 $$\underset{R_{c}\to \infty }{\lim}\int_{0}^{\infty }r^{d-1}dr\int_{\Omega_{d}}d{\mathrm{r̂}}_{ij}g\left(w_{p}\left(r,{\mathrm{r̂}}_{ij},{\mathrm{d̂}}_{i},{\mathrm{d̂}}_{j};R_{c},\epsilon \right)\right)=-C_{g}^{d}p^{2}{\mathrm{d̂}}_{i}\cdot {\mathrm{d̂}}_{j}f_{d}\left(\epsilon \right)\frac{\Omega_{d}}{d}+\Omega_{d}\int_{0}^{\infty }r^{d-1}dr\langle g\left(w_{p}\left(r,{\mathrm{r̂}}_{ij},{\mathrm{d̂}}_{i},{\mathrm{d̂}}_{j}\right)\right)\rangle_{{\mathrm{r̂}}_{ij}}$$

Equation 17 follows by owing to eq 16 and expanding *g* in a Taylor series in *w*_*p*_. Once using the mean reaction-field construction, each term of the expansion yields an absolutely convergent integral, allowing the exchange of the limit and the integration. The constant $C_{g}^{d}$ stems from the linear term in the Taylor expansion. As *d* → ∞, the first term in eq 17 vanishes.

## 3 Bulk Ferroelectricity of Dipolar Liquids in Large Dimensions

### 3.1 Virial Expansion and Density-Functional Theory

The virial expansion is a high-temperature expansion which can also be interpreted as a large *d* expansion.^20^ In the limit *d* → ∞, and under suitable conditions, the excess free energy of a hard-sphere liquid with respect to the ideal gas reduces to the second virial term, corresponding to direct two-particle interaction.^20^ In this limit, contributions to the free energy arising from correlations beyond the pair level vanish. This result is valid as long as the packing fraction of the liquid satisfies a suitable upper-bound condition,^20^ ensuring that the second virial coefficient remains finite. If this condition is violated, the virial expansion diverges and higher-order virial coefficients, associated with correlations beyond the pair level, become dominant. The dipolar interaction in eq 15, although anisotropic in **r**-space, is still rotationally invariant in the extended configuration space including both translational and dipolar degrees of freedom. The arguments of Ref. 20 establishing the exactness of the second-order truncation of the virial series can therefore be extended to the interaction potential in eq 15.^41^ Including dipolar degrees of freedom makes the radius of convergence of the virial expansion dependent on parameters associated with these additional degrees of freedom. The conditions of validity for the second-order truncation in the limit *d* → ∞ therefore translate into corresponding constraints on these parameters, as discussed below.

The central object of the virial expansion is the Mayer function. For the potential *v* in eq 2 with λ = 1, and the dipolar potential in eq 15, it reads

18 $$f\left(r,{\mathrm{r̂}}_{ij},{\mathrm{d̂}}_{i},{\mathrm{d̂}}_{j};R_{c},\epsilon \right)=e^{-\beta [v_{0}(r)+w_{p}(r,{\mathrm{r̂}}_{ij},{\mathrm{d̂}}_{i},{\mathrm{d̂}}_{j};R_{c},\epsilon )]}-1$$

The large-*d* limit of the corresponding free energy *F*[ρ̃] is obtained by truncating the virial expansion at second order and taking the limit *R*_*c*_ → ∞. One finds

19 $$-\beta F\left[\mathrm{\rho ̃}\right]=-\beta F^{id}\left[\mathrm{\rho ̃}\right]+\frac{N\rho }{2}\Omega_{d}\int r^{d-1}drd{\mathrm{d̂}}_{i}d{\mathrm{d̂}}_{j}\zeta \left({\mathrm{d̂}}_{i}\right)\langle f\left(r,{\mathrm{r̂}}_{ij},{\mathrm{d̂}}_{i},{\mathrm{d̂}}_{j}\right)\rangle_{{\mathrm{r̂}}_{ij}}\zeta \left({\mathrm{d̂}}_{j}\right)$$

where *F* ^*id*^[ρ̃] is the ideal-gas free energy. eq 19 follows from eq 17 by choosing *g* = *f*. Within the replicated liquid theory framework,^20^ eq 19 refers to the single-replica (liquid), and the average over *r̂*_*ij*_ corresponds to an annealed average. At second virial order, the excess free energy is a linear functional of *f*. This makes it straightforward to introduce an effective two-body screened dipolar potential obtained after carrying out the annealed average over *r̂*_*ij*_ in eq 19, as discussed in Sections 3.2–3.3. Equation 19 already provides a representation of the free energy in terms of the one-particle density field and therefore naturally falls within the DFT framework. It can be recast in the standard DFT expression, with the free energy decomposed into contributions from a reference system and a perturbative potential. Decomposing the Mayer function in eq 18 as

20 $$f\left(r,{\mathrm{r̂}}_{ij},{\mathrm{d̂}}_{i},{\mathrm{d̂}}_{j};R_{c},\epsilon \right)=f_{v_{0}}\left(r\right)+e^{-\beta v_{0}(r)}f_{w_{p}}\left(r,{\mathrm{r̂}}_{ij},{\mathrm{d̂}}_{i},{\mathrm{d̂}}_{j};R_{c},\epsilon \right)$$

with $f_{v_{0}}=e^{-\beta v_{0}}-1$ and $f_{w_{p}}=e^{-\beta w_{p}}-1$ , eq 19 reduces to

21 $$F\left[\mathrm{\rho ̃}\right]=F_{v_{0}}\left[\mathrm{\rho ̃}\right]+F\left[\mathrm{\rho ̃}\right]$$

$F_{v_{0}}\left[\mathrm{\rho ̃}\right]$ is the free energy of the reference system, describing particles interacting through *v*_0_ and carrying noninteracting dipoles, while F is the excess free energy associated with *w*_*p*_,

22 $$F\left[\mathrm{\rho ̃}\right]=-\frac{N\rho }{2\beta }\Omega_{d}\int r^{d-1}dre^{-\beta v_{0}(r)}d{\mathrm{d̂}}_{i}d{\mathrm{d̂}}_{j}\zeta \left({\mathrm{d̂}}_{i}\right)\langle f_{w_{p}}\left(r,{\mathrm{r̂}}_{ij},{\mathrm{d̂}}_{i},{\mathrm{d̂}}_{j}\right)\rangle_{{\mathrm{r̂}}_{ij}}\zeta \left({\mathrm{d̂}}_{j}\right)$$

eq 22 provides the exact DFT expression for the excess free energy in the limit *d* → ∞.

In the limit *d* → ∞, the entropic cost for breaking orientational isotropy scales as *O*(*d*), as shown in point (ii) of Appendix A. Therefore, in order for the dipolar interaction to produce a nontrivial contribution to the excess free energy and compete with the entropic term, its magnitude must scale accordingly. This is achieved by assuming

23 $$p^{2}=d{\mathrm{p̅}}^{2}$$

With this scaling the dipolar interaction remains finite only within a thin boundary layer around *r* = *l*, of thickness *O*(*l* log *d*/*d*), as shown in point (iii) of Appendix A, which is conveniently parametrized as,

24 $$r=l\left(1+\frac{\log d+h}{d}\right),,h=O\left(1\right)$$

In this region indeed it is

25 $$\beta p^{2}{\left(\frac{l}{r}\right)}^{d}\underset{d\to \infty }{\to }\beta {\mathrm{p̅}}^{2}e^{-h}$$

However, as shown in Appendix A(iii), under the scaling in eq 23 the dipolar interaction can become unbounded in the limit *d* → ∞ whenever *h* < 0 with sufficiently large magnitude, corresponding to the region $l<r<l\left(1+\frac{\log d}{d}\right)$ , possibly inducing instability in the system. As a consequence, the large-*d* limit is well-defined only outside this region. This can be implemented by introducing an effective core at $l_{\mathrm{eff}}=l\left(1+\frac{\log d}{d}\right)$ , which replaces *l* in the Heaviside function in eq 15, so that the condition *r* > *l*_eff_ becomes equivalent to *h* > 0, following eq 24. This directly yields the Heaviside function θ­(*h*) in eq 29. Finally, in order to obtain a nontrivial contribution from the isotropic potential *v*_0_(*r*), its amplitude is assumed to scale with *d*, as for the dipolar interaction so that under the radial scaling in eq 24 one obtains *v*_0_(*r*) → *v*_0_(*h*), with v_0_(h) finite for *d* → ∞. As detailed in Appendix A(iii), the excess free-energy in the limit *d* → ∞ thus reduces to

26 $$F\left[\mathrm{\rho ̃}\right]=N\rho B_{2}^{HS}\int d{\mathrm{d̂}}_{i}d{\mathrm{d̂}}_{j}\zeta \left({\mathrm{d̂}}_{i}\right)I\left({\mathrm{d̂}}_{i}\cdot {\mathrm{d̂}}_{j}\right)\zeta \left({\mathrm{d̂}}_{j}\right)$$

where $B_{2}^{HS}=\Omega_{d}l^{d}/\left(2d\right)$ is the hard-sphere second virial coefficient, and the reduced density $\rho B_{2}^{HS}$ is kept finite in the limit *d* → ∞. The kernel *I*(*d̂*_*i*_·*d̂*_*j*_) is given by

27 $$I\left({\mathrm{d̂}}_{i}\cdot {\mathrm{d̂}}_{j}\right)=-\frac{d}{\beta }\int_{-\infty }^{\infty }dhe^{h}e^{-\beta v_{0}(h)}{\mathrm{f̅}}_{w_{p}}\left({\mathrm{d̂}}_{i}\cdot {\mathrm{d̂}}_{j},h\right)$$

28 $${\mathrm{f̅}}_{w_{p}}\left({\mathrm{d̂}}_{i}\cdot {\mathrm{d̂}}_{j},h\right)=\underset{d\to \infty }{\lim}\langle f_{w_{p}}\left(r,{\mathrm{r̂}}_{ij},{\mathrm{d̂}}_{i},{\mathrm{d̂}}_{j}\right)\rangle_{{\mathrm{r̂}}_{ij}}$$

Making eq 28 explicit, see Appendix A(iii),

29 $${\mathrm{f̅}}_{w_{p}}\left({\mathrm{d̂}}_{i}\cdot {\mathrm{d̂}}_{j},h\right)=\underset{d\to \infty }{\lim}\frac{1}{\Omega_{d}}\int d{\mathrm{r̂}}_{ij}e^{-\beta {\mathrm{p̅}}^{2}\left[d\left({\mathrm{d̂}}_{i}\cdot {\mathrm{r̂}}_{ij}\right)\left({\mathrm{d̂}}_{j}\cdot {\mathrm{r̂}}_{ij}\right)-{\mathrm{d̂}}_{i}\cdot {\mathrm{d̂}}_{j}\right]\theta \left(h\right)e^{-h}}-1$$

Notice that, after averaging $f_{w_{p}}\left(r,{\mathrm{r̂}}_{ij},{\mathrm{d̂}}_{i},{\mathrm{d̂}}_{j}\right)$ over the uniform solid-angle distribution of *r̂*_*ij*_, the resulting function *f̅*(*r*, *d̂*_*i*_, *d̂*_*j*_) depends only on the scalar product *d̂*_*i*_·*d̂*_*j*_, as explicitly shown in Section 3.3. If the Mayer function ${\mathrm{f̅}}_{w_{p}}\left({\mathrm{d̂}}_{i}\cdot {\mathrm{d̂}}_{j},h\right)$ remains uniformly bounded, and *v*_0_ is tempered, as in the present case, the virial series terms of order *n* ≥ 3 are subleading in the limit *d* → ∞ at fixed reduced density $\rho B_{2}^{HS}=O\left(1\right)$ .^20^ The condition of uniformly boundedness of ${\mathrm{f̅}}_{w_{p}}\left({\mathrm{d̂}}_{i}\cdot {\mathrm{d̂}}_{j},h\right)$ imposes a threshold on β*p̅* ^2^, as shown in Section 3.3 and Appendix A(v) to which the validity of the second-order truncation of the virial series is therefore restricted. Under this condition, the standard large-*d* exactness proof for hard spheres^20^ extends straightforwardly to the potential *v*_0_ + *w*_*p*_. As can be inferred from eqs 26–29, the excess free energy in the limit *d* → ∞ retains a specific dependence on the dipole-orientation probability distribution, entirely encoded in ${\mathrm{f̅}}_{w_{p}}\left({\mathrm{d̂}}_{i}\cdot {\mathrm{d̂}}_{j},h\right)$ . Furthermore, the boundedness of ${\mathrm{f̅}}_{w_{p}}\left({\mathrm{d̂}}_{i}\cdot {\mathrm{d̂}}_{j},h\right)$ , implies that F=O(d) in the limit *d* → ∞.

Owing to the general identity

30 $$\frac{\delta F\left[\mathrm{\rho ̃}\right]}{\delta v\left(r_{i},r_{j},{\mathrm{d̂}}_{i},{\mathrm{d̂}}_{j}\right)}=-\frac{1}{2}\rho^{\left(2\right)}\left(r_{i},r_{j},{\mathrm{d̂}}_{i},{\mathrm{d̂}}_{j}\right)$$

it follows immediately that, upon truncating the virial expansion at second order and using the one-particle density field in eq 6, the pair correlation function reads

31 $$g^{\left(2\right)}\left(r,{\mathrm{r̂}}_{ij},{\mathrm{d̂}}_{i},{\mathrm{d̂}}_{j}\right)=e^{-\beta [v_{0}(r)+w_{p}(r,{\mathrm{r̂}}_{ij},{\mathrm{d̂}}_{i},{\mathrm{d̂}}_{j})]}$$

The same ansatz in eq 31 has been used in Refs 28,32,42–100 for dipolar liquids and in Refs 30,36,44–46 for classical Heisenberg fluids, and referred to as so-called modified mean-field approximation. In these studies, its use is justified in the low-density limit. In Ref 28, the use of the ansatz in eq 31 is shown to lead to a ferroelectric phase transition in dipolar liquids. However, the analysis is based on the bare dipolar interaction, without including an Onsager-like reaction-field construction, and the pair correlation function in eq 31 is expanded for small *p*, retaining only the leading terms. As a consequence, the dominant contribution to the free energy driving the ferroelectric phase transition originates from the surface term. The study therefore does not clarify whether the ferroelectric phase transition is a genuine bulk phenomenon. Furthermore, while the ansatz in eq 31 becomes exact as *d* → ∞, it remains approximate in three dimensions, as observed in Section 1.

### 3.2 Annealed Positional Disorder and the Emergence of Ferroelectricity

Equation 22 shows that, in the limit *d* → ∞, the free energy of the dipolar liquid is equivalent to that obtained from the effective pair Boltzmann factor

32 $$\langle e^{-\beta [v_{0}(r_{ij})+w_{p}(r_{ij},{\mathrm{r̂}}_{ij},{\mathrm{d̂}}_{i},{\mathrm{d̂}}_{j})]}\rangle_{{\mathrm{r̂}}_{ij}}$$

This follows directly from the exactness, in the large-*d* limit, of the truncation of the virial expansion at second order, implying that only pair interactions contribute to the free energy. Otherwise, the free energy would also contain annealed averages involving products of Boltzmann factors associated with different particle pairs. To clarify the role played by the annealed average over *r̂*_*ij*_ in the emergence of ferroelectricity in dipolar liquids in the large-*d* limit, it is useful to consider the expression of the liquid free energy in terms of the partition function, eq 11. Since, in the large-*d* limit, the free energy of the dipolar liquid is entirely determined by the effective Boltzmann factor in eq 32, the following relation holds:

$$Z\equiv \langle \underset{i<j}{\prod }e^{-\beta [\upsilon_{0}(r_{ij})+\omega_{p}(r_{ij},{\mathrm{r̂}}_{ij},{\mathrm{d̂}}_{i},{\mathrm{d̂}}_{j})]}\rangle_{\{r_{i},{\mathrm{d̂}}_{i}\}}\propto \langle \underset{i<j}{\prod }\langle e^{-\beta [\upsilon_{0}(r_{ij})+\omega_{p}(r_{ij},{\mathrm{r̂}}_{ij},{\mathrm{d̂}}_{i},{\mathrm{d̂}}_{j})]}\rangle_{{\mathrm{r̂}}_{ij}}\rangle_{\{r_{ij},{\mathrm{d̂}}_{i}\}}$$ 33

where the proportionality constant is physically irrelevant. In eq 33, the annealed average in the right-hand side could, in principle, be extended from *r̂*_*ij*_ to the full vectors **r**_*ij*_ without affecting the formal validity of the relation. However, the physical mechanism underlying the ferroelectric phase transition is encoded in the annealed average over *r̂*_*ij*_, which, through the functional form of *w*_*p*_, controls the effective dipole–dipole coupling. Nevertheless, the observation above emphasizes that this average only represents an intermediate mathematical step introduced to isolate the physically relevant features of the problem. It is not an approximation and, in particular, it does not rely on any separation of time scales between translational and dipolar degrees of freedom.

It is instructive to draw an analogy with the annealed counterpart of the Sherrington–Kirkpatrick spin-glass model.^47^ Let a system of interacting vector spins *d̂*_*i*_ with Hamiltonian

34 $$H=\underset{i<j}{\sum }J_{ij}{\mathrm{d̂}}_{i}\cdot {\mathrm{d̂}}_{j}$$

where the couplings *J*_*ij*_ are independently sampled from a zero-mean Gaussian distribution. The partition function of the annealed system can be written as

35 $$Z\equiv \langle \underset{i<j}{\prod }e^{-\beta J_{ij}{\mathrm{d̂}}_{i}\cdot {\mathrm{d̂}}_{j}}\rangle_{\{J_{ij},{\mathrm{d̂}}_{i}\}}\propto \langle \underset{i<j}{\prod }\langle e^{-\beta J_{ij}{\mathrm{d̂}}_{i}\cdot {\mathrm{d̂}}_{j}}\rangle_{J_{ij}}\rangle_{\{{\mathrm{d̂}}_{i}\}}$$

where $\langle \rangle_{\{J_{ij},{\mathrm{d̂}}_{i}\}}$ is the ensemble average over both coupling realizations and spin configurations, $\langle \rangle_{J_{ij}}$ is the average over the distribution of *J*_*ij*_, and $\langle \rangle_{\{{\mathrm{d̂}}_{i}\}}$ is the ensemble average over spin configurations. In this case, eq 35 follows from the pairwise structure of the Hamiltonian and from the statistical independence of the couplings *J*_*ij*_. The analogy with eq 33 is immediate, with *r̂*_*ij*_ playing a role formally analogous to that of the random couplings *J*_*ij*_. Interestingly, the annealed Sherrington–Kirkpatrick spin-glass model is known to exhibit a hidden Mattis phase with spin order.^48,49^

Equations 22 and 33 show that, in the limit *d* → ∞, a screened dipolar potential *W̅* satisfying eq 12 can be defined through the annealed average of the dipolar potential *w*_*p*_ over *r̂*_*ij*_. It reads as

36 $${\mathrm{W̅}}_{d\to \infty }\left(r,{\mathrm{d̂}}_{i},{\mathrm{d̂}}_{j}\right)=-\frac{1}{\beta }\underset{d\to \infty }{\lim}\log\langle e^{-\beta w_{p}(r,{\mathrm{r̂}}_{ij},{\mathrm{d̂}}_{i},{\mathrm{d̂}}_{j})}\rangle_{{\mathrm{r̂}}_{ij}}$$

To address the issues raised in Section 1 the remaining step is to determine whether the screened potential *W̅* in eq 36 is ferroelectric-like, according to the definition introduced in Section 1, and short-ranged. Before proceeding to a fully quantitative analysis, useful qualitative insight can be obtained by considering the *d*-dimensional generalization of an octahedral quadrature scheme. Within this approximation,

37 $$e^{-\beta {\mathrm{W̅}}_{oq,d}(r,{\mathrm{d̂}}_{i},{\mathrm{d̂}}_{j})}=\frac{1}{2d}\overset{d}{\underset{\alpha =1}{\sum }}\underset{\sigma =\pm 1}{\sum }e^{-\beta w_{p}(r,\sigma {ê}_{k},{\mathrm{d̂}}_{i},{\mathrm{d̂}}_{j})}$$

where $\{{ê}_{k}{\}}_{\alpha =1}^{d}$ denotes the canonical basis of $R^{d}$ . The sum runs over the 2*d* vertices of the *d*-dimensional cross-polytope. Choosing one quadrature direction parallel to *d̂*_*i*_, eq 37 reduces to

38 $$e^{-\beta {\mathrm{W̅}}_{oq,d}(r,{\mathrm{d̂}}_{i},{\mathrm{d̂}}_{j})}=\frac{1}{2d}\left[2e^{\beta (d-1)p^{2}{\left(\frac{l}{r}\right)}^{d}{\mathrm{d̂}}_{i}\cdot {\mathrm{d̂}}_{j}}+2\left(d-1\right)e^{-\beta p^{2}{\left(\frac{l}{r}\right)}^{d}{\mathrm{d̂}}_{i}\cdot {\mathrm{d̂}}_{j}}\right]$$

In the large-*d* limit,

39 $${\mathrm{W̅}}_{oq,d\to \infty }\left(r,{\mathrm{d̂}}_{i},{\mathrm{d̂}}_{j}\right)∼-\frac{1}{\beta }\log\left[2e^{\beta (d-1)p^{2}{\left(\frac{l}{r}\right)}^{d}{\mathrm{d̂}}_{i}\cdot {\mathrm{d̂}}_{j}}+2\left(d-1\right)e^{-\beta p^{2}{\left(\frac{l}{r}\right)}^{d}{\mathrm{d̂}}_{i}\cdot {\mathrm{d̂}}_{j}}\right]$$

where the additive term −log­(2*d*) has been omitted, since it is orientation-independent. Minimizing the effective potential is equivalent to maximizing the quantity inside square brackets in eq 39. The first term is exponentially increasing with *d̂*_*i*_·*d̂*_*j*_, whereas the second is exponentially decreasing. In the large-*d* limit, the growth of the first term dominates, and the maximum is therefore attained for *d̂*_*i*_·*d̂*_*j*_ = 1, implying that ${\mathrm{W̅}}_{oq}^{\left(d\right)}$ is minimized in the ferroelectric configuration. In the limit *d* → ∞ the minimum of *W̅* at *d̂*_*i*_·*d̂*_*j*_ = 1 is accompanied by an unphysical divergence. This originates from the octahedral quadrature, which does not correctly reproduce the large-*d* solid-angle measure and assigns finite weight to angular regions whose measure vanishes in the limit *d* → ∞.

The logarithm in eq 36 can be expanded as log­(1+*x*) = *x*+O­(*x* ^2^), with $x=\langle e^{-\beta w_{p}}\rangle_{{\mathrm{r̂}}_{ij}}-1=-\beta \langle w_{p}\rangle_{{\mathrm{r̂}}_{ij}}+\frac{\beta^{2}}{2}\langle w_{p}^{2}\rangle_{{\mathrm{r̂}}_{ij}}+\cdot \cdot \cdot$ . Since $\langle w_{p}\rangle_{{\mathrm{r̂}}_{ij}}=0$ , as shown in Section. [Sec sec1], the leading contribution is therefore proportional to $\langle w_{p}^{2}\rangle_{{\mathrm{r̂}}_{ij}}$ . This indeed anticipates that the screened potential *W̅* is shorter-ranged than the bare dipolar potential *w*_*p*_.

### 3.3 On the Onset of Ferroelectricity: An Exact Result

As shown in Section 3.1 the quantity ${\mathrm{f̅}}_{w_{p}}\left({\mathrm{d̂}}_{i}\cdot {\mathrm{d̂}}_{j},h\right)$ defined in eq 29 fully determines F in the limit *d* → ∞. Once ${\mathrm{f̅}}_{w_{p}}\left({\mathrm{d̂}}_{i}\cdot {\mathrm{d̂}}_{j},h\right)$ is explicitly evaluated, the free energy is completely specified as a functional of the one-particle density field ρζ­(*d̂*). Minimization of the free energy with respect to ζ­(*d̂*) determines whether ferroelectric order can set in. This is the task addressed in the following.

Changing variables from *r̂*_*ij*_ to **t**_*ij*_ = (θ_*i*_, θ_*j*_) = (*d̂*_*i*_·*r̂*_*ij*_, *d̂*_*j*_·*r̂*_*ij*_), one obtains

40 $$\langle f_{w_{p}}\left(r,{\mathrm{r̂}}_{ij},{\mathrm{d̂}}_{i},{\mathrm{d̂}}_{j}\right)\rangle_{{\mathrm{r̂}}_{ij}}=\frac{\Omega_{d-2}}{\Omega_{d}}\int_{D}\frac{dt_{ij}}{{\left(\det G\right)}^{1/2}}{\left(1-t_{ij}G^{-1}t_{ij}^{T}\right)}^{d-4/2}e^{-\beta p^{2}{(\frac{l}{r})}^{d}\left[{\mathrm{d̂}}_{i}\cdot {\mathrm{d̂}}_{j}-d\theta_{i}\theta_{j}\right]}-1$$

where $D=\left\{(t_{ij}:t_{ij}G^{-1}t_{ij}^{T}\leq 1\right\}$ , $t_{ij}^{T}$ is the transpose of **t**_*ij*_, and *G* is the (2 × 2) Gram matrix with entries *G*_*ij*_ = *d̂*_*i*_·*d̂*_*j*_. Details are given in Appendix A(v). Eq 40 shows that $\langle f_{w_{p}}\left(r,{\mathrm{r̂}}_{ij},{\mathrm{d̂}}_{i},{\mathrm{d̂}}_{j}\right)\rangle_{{\mathrm{r̂}}_{ij}}$ depends on *d̂*_*i*_ and *d̂*_*j*_ only through their scalar product, as anticipated in Section 3.1. To evaluate the limit *d* → ∞ it is convenient to introduce the rescaled variable ${\mathrm{t̃}}_{ij}=\sqrt{d}t_{ij}$ . As in Appendix A(v), in the limit *d* → ∞ one obtains

$$\frac{\Omega_{d-2}}{\Omega_{d}}\frac{1}{{\left(\det G\right)}^{1/2}}{\left(1-t_{ij}G^{-1}t_{ij}^{T}\right)}^{d-4/2}dt_{ij}\underset{d\to \infty }{\to }\frac{1}{2\pi {\left(\det G\right)}^{1/2}}e^{-\frac{1}{2}{\mathrm{t̃}}_{ij}G^{-1}{\mathrm{t̃}}_{ij}^{T}}d{\mathrm{t̃}}_{ij}$$ 41

with $\Omega_{d}=\frac{{2\pi }^{d/2}}{\Gamma \left(\frac{d}{2}\right)}$ , and Γ­() the Euler Gamma function. The probability distribution of the rescaled variables **t̃**_*ij*_, induced by the uniform measure on *r̂*_*ij*_, therefore converges for *d* → ∞ to a centered bivariate Gaussian distribution with covariance matrix **G**. Using the scaling variable *h* for *r*, see eq 29, one finally obtains

42 $${\mathrm{f̅}}_{w_{p}}\left({\mathrm{d̂}}_{i}\cdot {\mathrm{d̂}}_{j},h\right)=\int_{-\infty }^{\infty }d{\mathrm{t̃}}_{ij}\frac{e^{-\frac{1}{2}{\mathrm{t̃}}_{ij}G^{-1}{\mathrm{t̃}}_{ij}^{T}}}{2\pi {\left(\det G\right)}^{1/2}}e^{-\beta {\mathrm{p̅}}^{2}\left[{\mathrm{d̂}}_{i}\cdot {\mathrm{d̂}}_{j}-{\mathrm{\theta ̃}}_{i}{\mathrm{\theta ̃}}_{j}\right]\theta \left(h\right)e^{-h}}-1$$

The integral in eq 42 converges for all *h* ≥ 0 provided $\beta {\mathrm{p̅}}^{2}<\frac{1}{2}$ , thereby identifying the necessary condition for the second-order truncation of the virial series to be exact in the limit *d* → ∞. For sufficiently large values of *p̅* higher-order orientational correlations among dipoles indeed emerge, and through the dipolar interaction, induce effective correlations among particle positions. Higher-order terms in the virial expansion become thus dominant. The integral in eq 42 coincides with the moment-generating function *M*_*Z̃*_(*t*) of the random variable

43 $$\mathrm{Z̃}=\left[{\mathrm{\theta ̃}}_{i}{\mathrm{\theta ̃}}_{j}-{\mathrm{d̂}}_{i}\cdot {\mathrm{d̂}}_{j}\right]$$

evaluated with respect to the bivariate Gaussian distribution of **t**_*ij*_ defined in eq 41. Eq 42 can thus be written as

44 $${\mathrm{f̅}}_{w_{p}}\left({\mathrm{d̂}}_{i}\cdot {\mathrm{d̂}}_{j},h\right)=\langle e^{t\mathrm{Z̃}}\rangle_{{\mathrm{r̂}}_{ij}}-1=M_{\mathrm{Z̃}}\left(t\right)-1$$

with *t* = β*p̅* ^2^θ­(*h*) e^–*h*^ and therefore $t<\frac{1}{2}$ under the convergence condition. As shown in Appendix A(v),

45 $$M_{\mathrm{Z̃}}\left(t\right)=e^{-t{\mathrm{d̂}}_{i}\cdot {\mathrm{d̂}}_{j}-\frac{1}{2}\log[1-2t{\mathrm{d̂}}_{i}\cdot {\mathrm{d̂}}_{j}-t^{2}\left(1-{{\mathrm{d̂}}_{i}\cdot {\mathrm{d̂}}_{j}}^{2}\right)]}$$

Because $\langle w_{p}\rangle_{{\mathrm{r̂}}_{ij}}=0,\langle \mathrm{Z̃}\rangle_{{\mathrm{r̂}}_{ij}}=0$ , and Jensen’s inequality^50^ then implies $M_{\mathrm{Z̃}}\left(t\right)=\langle e^{t\mathrm{Z̃}}\rangle_{{\mathrm{r̂}}_{ij}}\geq e^{t\langle \mathrm{Z̃}\rangle_{{\mathrm{r̂}}_{ij}}}=1$ , ∀ *t*. From eqs 26–29 and 44–45, minimizing F then amounts to maximizing *M*_*Z̃*_(*t*). A direct calculation from eq 45 shows that *M*_*Z̃*_(*t*), as a function of *d̂*_*i*_·*d̂*_*j*_, attains its maximum at *d̂*_*i*_·*d̂*_*j*_ = 1. This behavior is readily confirmed by inspection of Figure 3, which displays ${\mathrm{f̅}}_{w_{p}}\left({\mathrm{d̂}}_{i}\cdot {\mathrm{d̂}}_{j},t\right)=M_{\mathrm{Z̃}}\left(t\right)-1$ as a function of ${\mathrm{d̂}}_{i}\cdot {\mathrm{d̂}}_{j}$ for different values of *t*. In particular, for fixed β and *h*, increasing values of *t* correspond to increasing values of the dipole moment *p̅*. F is therefore minimized by ζ­(*d̂*) in which all dipoles align along a common direction. On the other hand, $F_{v_{0}}\left[\mathrm{\rho ̃}\right]$ includes the orientational entropy of noninteracting dipoles, which is maximized by an isotropic orientational distribution. The competition between this entropic contribution and the excess free-energy F , whose balance is tuned by the thermodynamic parameters of the liquid, determines the onset of the ferroelectric phase transition in the dipolar liquid.^13^ To make the discussion more quantitative, the simplified ansatz

46 $$\zeta \left(\mathrm{d̂}\right)=\frac{1}{Z_{d}\left(\delta \right)}e^{d\delta \cdot \mathrm{d̂}}$$

is considered, with *Z*_*d*_(**δ**) = ∫d*d̂* e ***^dδ^*** *^·d̂^*, and δ=δδ̂ with 0 ≤ δ ≤ 1. As shown in Appendix A-(i), in the limit *d* → ∞ this ansatz is able to discriminate between the paraelectric and ferroelectric phases. In particular,

$$\begin{array}{r} \mathrm{p̅}=\mathrm{p̅}\mathrm{u̅}\left(\delta \right)\mathrm{\delta ̂}\\ \mathrm{u̅}\left(\delta \right)=\left(\sqrt{1+4\delta^{2}}-1\right)/\left(2\delta \right) \end{array}$$ 47

For small δ, eq 47 yields ***p̅*** ∝ **δ**, showing that **δ** plays the role of the ferroelectric order parameter. The entropy difference between the dipole-ordered ferroelectric state (**δ** ≠ 0) and the dipole-isotropic paraelectric state (**δ** = 0), as shown in Appendix A(ii), is

48 $$\Delta S\left(\delta \right)=-Nd\left[\frac{1}{2}|\log\left(1-{\mathrm{u̅}}^{2}\left(\delta \right)\right)|\right]+o\left(d\right)$$

Here and throughout, |*x*| denotes the absolute value of *x*. It follows

49 $$\Delta F_{\mathrm{ent}}\left(\delta \right)=-\frac{1}{\beta }\Delta S\left(\delta \right)>0$$

The excess free-energy difference between ferroelectric and paraelectric state in the large-*d* limit is, as derived in Appendix A(iv),

50 $$\begin{array}{r} \Delta F\left(\rho ,\delta \right)=\frac{N\rho }{\beta }B_{2}^{HS}d\int_{-\infty }^{\infty }dhe^{h}e^{-\beta v_{0}(h)}f_{\delta }\left(h\right)\\ f_{\delta }\left(h\right)=-{\mathrm{f̅}}_{w_{p}}\left({\mathrm{u̅}}^{2}\left(\delta \right),h\right) \end{array}$$

with *u̅*(δ) given in eq 47. Because ${\mathrm{f̅}}_{w_{p}}\left({\mathrm{d̂}}_{i}\cdot {\mathrm{d̂}}_{j},h\right)\geq 0$ , *f*_δ_ < 0, ∀ *t*, see also Figure 3. As a consequence, a finite value of δ lowers F . Observe that, according to eqs 48–50, both Δ*F*_ent_(**δ**) and ΔF(ρ,δ) scale as *O*(*Nd*). While the former is strictly positive and therefore penalizes orientational ordering, the latter is negative and favors the onset of ferroelectric order. The dependence of ΔF on the density has been made explicit. Moreover, as follows from eq 50, ΔF also depends on temperature. The thermodynamic parameters can therefore drive the quantity $\Delta F_{\mathrm{ent}}\left(\delta \right)+\Delta F\left(\rho ,\delta \right)$ from positive to negative values, inducing the ferroelectric phase transition. A Taylor expansion of the two contributions for small δ yields a Landau-like free-energy expression in terms of the ferroelectric order parameter. Similar developments are presented in Ref 13, where the interplay between ferroelectric and liquid–liquid phase transitions is also discussed in detail, and are beyond the scope of the present study. As emphasized in Ref 13, the emergence of a ferroelectric phase transition relies on the negative sign of ΔF(δ) . The present analysis identifies the physical origin of this negative contribution, showing it to be a direct consequence of the presence of annealed positional disorder.

**Figure 3:**
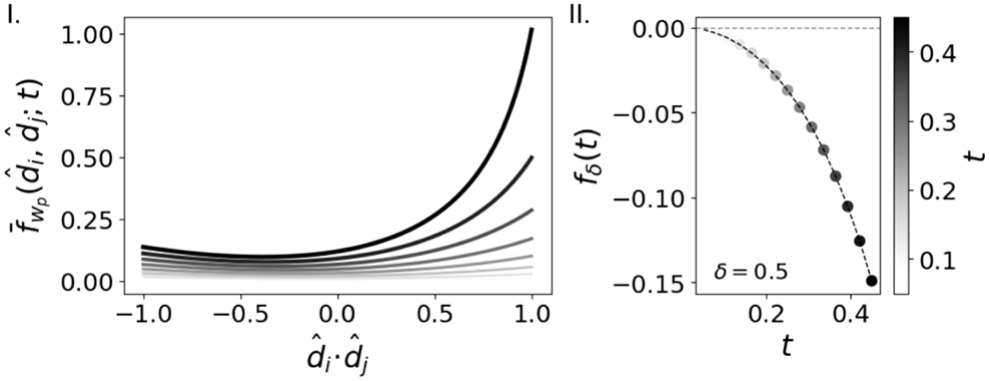
*Panel I.* ${\mathrm{f̅}}_{w_{p}}\left({\mathrm{d̂}}_{i}\cdot {\mathrm{d̂}}_{j},t\right)$ as a function of *d̂_i_* ·*d̂_j_* for different values of *t*. Increasing *t* leads to progressively larger bias toward *d̂_i_* ·*d̂_j_* = 1, indicating an enhanced ferroelectric-like character of *W̅_d→∞_*. At fixed β and *h*, larger values of *t* correspond to larger values of p̅. *Panel II.* Corresponding values of *f*_δ_ as a function of *t*. ∀ *t*, *f*_δ_ < 0.

### 3.4 Short-Range Annealed-Averaged Dipolar Interaction: Hindered Dipolar Rotation and Pair Correlations

Once the explicit expression of ${\mathrm{f̅}}_{w_{p}}\left(r,{\mathrm{d̂}}_{i}\cdot {\mathrm{d̂}}_{j}\right)$ is known, see eq 44 and 45, the effective potential *W̅*_*d→∞*_ in eq 36 can be obtained in closed form,

51 $${\mathrm{W̅}}_{d\to \infty }=\frac{1}{\beta }\left[t{\mathrm{d̂}}_{i}\cdot {\mathrm{d̂}}_{j}+\frac{1}{2}\log\left[1-2t{\mathrm{d̂}}_{i}\cdot {\mathrm{d̂}}_{j}-t^{2}\left(1-{{\mathrm{d̂}}_{i}\cdot {\mathrm{d̂}}_{j}}^{2}\right)\right]\right],,t=\beta p^{2}\theta \left(h\right)e^{-h}$$

In the following, the effective interaction *W̅*_d→∞_ is shown to be shorter-ranged than the bare dipolar potential *w*_*p*_, as a consequence of the screening induced by annealed positional disorder. This mechanism mirrors the screening arising from annealed averaging upon freely rotating dipoles, which leads to the Keesom interaction. From eqs 36, and 44, eq 51 can be rewritten as

52 $${\mathrm{W̅}}_{d\to \infty }=-\frac{1}{\beta }K_{\mathrm{Z̃}}\left(t\right)$$

where K_*Z̃*_(*t*) = log *M*_*Z̃*_(*t*) is the cumulant generating function associated with the random variable *Z̃*. Using the cumulant expansion

53 $$K_{\mathrm{Z̃}}\left(t\right)=\overset{\infty }{\underset{n=1}{\sum }}\frac{1}{n!}k_{\mathrm{Z̃}}^{\left(n\right)}t^{n},,k_{\mathrm{Z̃}}^{\left(n\right)}={\frac{d^{n}}{dt^{n}}K_{\mathrm{Z̃}}\left(t\right)|}_{t=0}$$

eq 52 becomes

54 $${\mathrm{W̅}}_{d\to \infty }=-\frac{1}{\beta }\overset{\infty }{\underset{n=2}{\sum }}\frac{1}{n!}{\left(\beta {\mathrm{p̅}}^{2}\right)}^{n}\theta \left(h\right)e^{-nh}k_{\mathrm{Z̃}}^{\left(n\right)}$$

The leading nonvanishing contribution in eq 54 arises at *n* = 2, since $k_{\mathrm{Z̃}}^{\left(1\right)}={\frac{d}{dt}\log\langle e^{t\mathrm{Z̃}}\rangle_{{\mathrm{r̂}}_{ij}}|}_{t=0}={\langle \mathrm{Z̃}e^{t\mathrm{Z̃}}\rangle_{{\mathrm{r̂}}_{ij}}/\left\langle e^{t\mathrm{Z̃}}\rangle_{{\mathrm{r̂}}_{ij}}\right|}_{t=0}=\langle \mathrm{Z̃}\rangle_{{\mathrm{r̂}}_{ij}}=0$ . *W̅* thus scales as e^–2*h*^ + *o*(e^–2*h*^), which, upon reverting from *h* to the radial coordinate *r*, yields

55 $${\mathrm{W̅}}_{d\to \infty }\left(r\right)\propto r^{-2d}+o\left(r^{-2d}\right)$$

Therefore, *W̅*_*d→∞*_ is shorter-ranged than the bare dipolar interaction.

*W̅*_*d→∞*_ has a ferroelectric-like character, as shown in Section 3.3. Following the discussion in Section 1 this is expected to result in a positive value of ⟨*d̂*_*i*_·*d̂*_*j*_⟩ even in the paraelectric phase. Eq 13 can be recast as

56 $$\langle {\mathrm{d̂}}_{i}\cdot {\mathrm{d̂}}_{j}\rangle_{\delta =0}=\frac{1}{Z_{0}}\int_{-\infty }^{\infty }dhe^{h}e^{-\beta v_{0}(h)}\int_{-1}^{1}dqqp_{d}\left(q\right)e^{-\beta {\mathrm{W̅}}_{d\to \infty }(h,q)}$$

where

57 $$q={\mathrm{d̂}}_{i}\cdot {\mathrm{d̂}}_{j}$$

and $Z_{0}=\int_{-\infty }^{\infty }dhe^{h}e^{-\beta v_{0}(h)}\int_{-1}^{1}dqp_{d}\left(q\right)e^{-\beta \mathrm{W̅}(h,q)}$ . The probability density of *q* for two independent unit vectors *d̂*_*i(j)*_ uniformly distributed in $R^{d}$ is

58 $$p_{d}\left(q\right)=\frac{\Omega_{d-1}}{\Omega_{d}}{\left(1-q^{2}\right)}^{d-3/2}$$

as obtained by the same argument used in Appendix A-(i). Since *W̅*_*d→∞*_ remains finite, while *p*_*d*_(*q*) becomes increasingly peaked around *q* = 0 for large *d*, eq 56 implies lim_d→∞_⟨*d̂*_*i*_·*d̂*_*j*_⟩ = 0. Although exact in the limit *d* → ∞, this result does not necessarily capture the behavior at large but finite dimensionality when approaching the *d* → ∞ limit, where the functional form of *W̅*_d_ may still significantly affect dipolar correlations. Increasing *d* primarily suppresses the magnitude of ⟨*d̂*_*i*_·*d̂*_*j*_⟩ through *p*_*d*_(*q*), without altering the ferroelectric character of *W̅_d_*. To isolate the effect of *W̅*_*d→∞*_ on hindered dipolar rotations, the case of a uniform angular measure *p*_*d*_(*q*) in eq 56 is analyzed below. Figure 4 reports: (i) the canonical probability distribution of *q* = *d̂*_*i*_·*d̂*_*j*_ associated with *W̅*_*d→∞*_, $P_{\mathrm{W̅}}\left(q|t\right)=e^{-\beta {\mathrm{W̅}}_{d\to \infty }}/Z_{\mathrm{W̅}}$ with $Z_{\mathrm{W̅}}=\int_{-1}^{1}dqe^{-\beta {\mathrm{W̅}}_{d\to \infty }(h,q)}$ ; (ii) the corresponding first spherical-harmonic moment (l=1) , ⟨*q̂*⟩_*W̅*_, measuring local dipolar alignment; and (iii) the second spherical-harmonic moment (l=2) , ⟨*P*_2_(*q*)⟩_*W̅*_, quantifying local quadrupolar (nematic-like) ordering. All quantities refer to the paraelectric phase, since eq 13 implicitly assumes ζ­(*d̂*) to be uniform over the solid angle. Figure 4 confirms that ⟨*q*⟩_*W̅*_ > 0, ∀ *t*. This follows from the ferroelectric character of *W̅*_*d→∞*_, which favors parallel dipolar alignment, as directly illustrated by Panel I of Figure 4, where *P*_*W̅*_(*q*|*t*) is biased toward positive values of *q*. These results show that a positive value of the dipolar correlation ⟨*d̂*_*i*_·*d̂*_*j*_⟩ in the paraelectric phase, readily accessible in numerical simulations, already signal the tendency of the system to develop a ferroelectric phase transition. If the first moment of *P*_*W̅*_(*q*|*t*), ⟨*d̂*_*i*_·*d̂*_*j*_⟩, already captures the qualitative ferroelectric-like character of *W̅*, higher-order moments are expected to provide a more quantitative characterization. They could in principle be exploited to reconstruct *P*_*W̅*_(*q*|*t*). In numerical simulations, this can be achieved by computing a finite set of moments and solving the associated inverse problem, for instance via maximum-entropy methods.^51^ By exploiting the equivalence, pointed out in Section 1, between *P*_*W̅*_(*q*|*t*) and the pair correlation function *g* ^(2)^ entering the DFT expression of F , see e.g., eq 4, this approach can thus provide direct numerical access to *g* ^(2)^. This, in turn, would enable identification of the onset of the ferroelectric phase transition and of the critical temperature from numerical simulations performed entirely within the paraelectric phase, providing an alternative to numerical simulation studies across the putative critical temperature, which rely on demanding finite-size scaling analyses. A different strategy is presented in Ref 52, where the DFT free-energy functional is learned directly from numerical simulation data using a neural-network approach.

**Figure 4:**
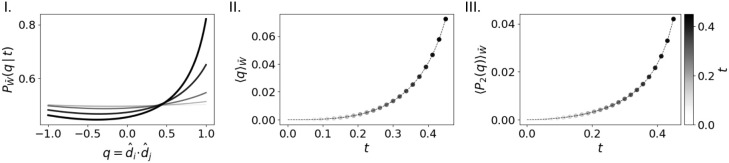
Boltzmann probability distribution associated with the effective potential *W̅*, *P_W̅_* (*q*|*t*) (*Panel I*) together with the corresponding first spherical-harmonic moment, ⟨*q*⟩_*w̅*_ (*Panel II*), and second spherical-harmonic moment ⟨*P*_2_(*q*)⟩_*W̅*_. All quantities are shown for different values of the parameter *t*. The second-order Legendre polynomial is defined as *P*_2_(*q*) = *dq* ^2^ – 1, evaluated here for *d* = 3.

Following eq 14, in the ferroelectric phase and in the limit *d* → ∞, it is

59 $$\langle {\mathrm{d̂}}_{i}\cdot {\mathrm{d̂}}_{j}\rangle_{\delta }=\frac{1}{Z_{\delta }}\int_{-\infty }^{\infty }dhe^{h}e^{-\beta v_{0}(h)}\int d{\mathrm{d̂}}_{i}d{\mathrm{d̂}}_{j}\zeta \left({\mathrm{d̂}}_{i}\right)\zeta \left({\mathrm{d̂}}_{j}\right){\mathrm{d̂}}_{i}\cdot {\mathrm{d̂}}_{j}e^{-\beta {\mathrm{W̅}}_{d\to \infty }(h,{\mathrm{d̂}}_{i}\cdot {\mathrm{d̂}}_{j})}$$

where $Z_{\delta }=\int_{-\infty }^{\infty }dhe^{h}e^{-\beta v_{0}(h)}\int d{\mathrm{d̂}}_{i}d{\mathrm{d̂}}_{j}\zeta \left({\mathrm{d̂}}_{i}\right)\zeta \left({\mathrm{d̂}}_{j}\right)e^{-\beta {\mathrm{W̅}}_{d\to \infty }(h,{\mathrm{d̂}}_{i}\cdot {\mathrm{d̂}}_{j})}$ and ζ­(*d̂*) is given in eq 46 with **δ** ≠ 0. The same steps used in Appendix A(iv), based on the Laplace saddle-point method, readily show that

60 $$\langle {\mathrm{d̂}}_{i}\cdot {\mathrm{d̂}}_{j}\rangle_{\delta }={\mathrm{u̅}}^{2}\left(\delta \right)={\mathrm{p̅}}^{2}$$

where *u̅*(δ) is given in eq 47. Since, as shown above, in the limit *d* → ∞, ⟨*d̂*_*i*_·*d̂*_*j*_⟩_δ = 0_ = 0 in the paraelectric phase, eq 60 suggests that the dipolar correlation ⟨*d̂*_*i*_·*d̂*_*j*_⟩ measured in numerical simulations may provide an indicator of the onset of the ferroelectric phase transition. In finite *d*, however, ⟨*d̂*_*i*_·*d̂*_*j*_⟩ retains a local contribution even within the paraelectric phase, which becomes progressively suppressed as *d* → ∞, as emphasized above. If using ⟨*d̂*_*i*_·*d̂*_*j*_⟩ as a diagnostic of ferroelectric ordering, it is important to recall that the hallmark of the ferroelectric phase transition is the emergence of a macroscopic polarization in the ordered phase. Eq 1 indeed implies that $\underset{N\to \infty }{\lim}\frac{1}{N^{2}}\overset{N}{\underset{i,j=1}{\sum }}\left\langle {\mathrm{d̂}}_{i}\cdot {\mathrm{d̂}}_{j}\right\rangle$ is *O*(1) in the ferroelectric phase, while it vanishes in the paraelectric phase. Distinguishing between the ferroelectric and paraelectric phases therefore requires finite-size scaling analyses in numerical simulations to determine whether ⟨*d̂*_*i*_·*d̂*_*j*_⟩ exhibits different scaling behavior in the two phases.

The emergence of ferroelectric order also leaves a clear signature on the radial pair correlation function marginalized over the dipolar degrees of freedom, ρ^(2)^(*r*), which can therefore serve as an indicator of the onset of the ferroelectric phase transition. Starting from eq 31, using the definition of *W̅*_*d*→∞_ in eq 36, the one-particle density in eq 6 and the scaling variable *h*, one obtains

61 $$\frac{\rho^{\left(2\right)}\left(h\right)}{\rho^{2}}=e^{-\beta v_{0}(h)}\int d{\mathrm{d̂}}_{i}d{\mathrm{d̂}}_{j}\zeta \left({\mathrm{d̂}}_{i}\right)\zeta \left({\mathrm{d̂}}_{j}\right)e^{-\beta {\mathrm{W̅}}_{d\to \infty }(h,{\mathrm{d̂}}_{i}\cdot {\mathrm{d̂}}_{j})}$$

Using for ζ­(*d̂*) the ansatz in eq 46, eq 61 reduces to

62 $$\frac{\rho_{\delta }^{\left(2\right)}\left(h\right)}{\rho^{2}}=e^{-\beta v_{0}(h)}e^{-\beta {\mathrm{W̅}}_{d\to \infty }(h,{\mathrm{u̅}}^{2}\left(\delta \right))}$$

where the Laplace method has been exploited. The derivation follows the same steps used in Appendix A(iv), in particular eqs 127–130. Equation 62 applies to both ferroelectric and paraelectric phases. In the latter case, δ = 0, which implies *p̅* = *u̅*(δ) = 0. Figure 5 shows the radial pair correlation $\rho_{\delta }^{\left(2\right)}\left(h\right)/\rho^{2}$ in the paraelectric (*p̅* = 0, full line) and ferroelectric phase (*p̅* = 0.69, dashed line). Lennard-Jones potential is chosen for the reference potential, so that *v*_0_(*h*) = *E*_0_(e*^–νh^* – e^–*νh*/2^),^20^ with ν = 4. Compared to the paraelectric phase, the characteristic peak of the pair correlation function in the ferroelectric phase is enhanced and slightly shifted toward smaller values of *h*, corresponding to reduced characteristic interparticle distances. This indicates that ferroelectric ordering promotes not only orientational correlations, but also local positional ordering, increasing the probability of finding particles at the same characteristic distance associated with the peak. The shift toward shorter distances can be understood intuitively by noting that, within a mean-field picture in which *pd̂*_*i*_ = *pd̂*_*j*_ = ***p̅***, the annealed averaged dipolar potential is attractive as a function of the interparticle distance *r*. This result is noteworthy because ρ^(2)^(*r*)/ρ^2^ coincides with the radial pair correlation function *g* ^(2)^(*r*) measured in numerical simulations when dipolar degrees of freedom are not explicitly resolved. In real fluids, *g* ^(2)^(*r*) typically exhibits multiple coordination shells. Nevertheless, this simplified scenario highlights an important mechanism: dipolar ordering alone can promote local spatial ordering, leaving clear signatures in the radial correlation function *g* ^(2)^(*r*). This observation may provide a useful perspective for interpreting changes observed in *g* ^(2)^(*r*) between the high-density and low-density phases of supercooled water.^53^ Moreover, it offers further consistency with associating a paraelectric character to the high-density and a ferroelectric character to the low-density phase.

**Figure 5:**
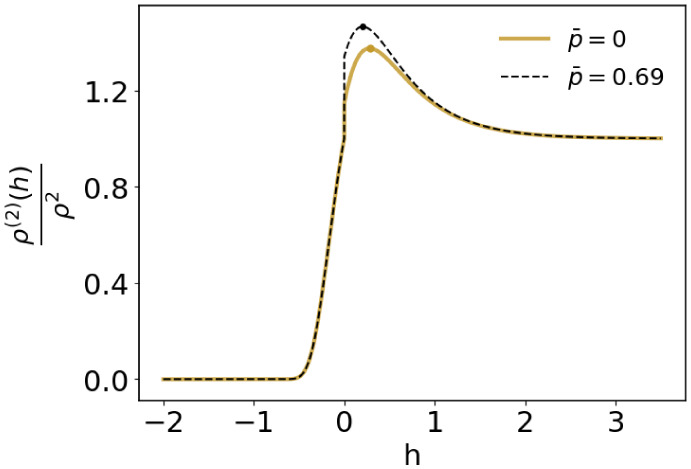
Radial pair correlation marginalized over dipolar degrees of freedom, $\frac{\rho^{\left(2\right)}}{\rho^{2}}$ , in the paraelectric (**p̅** = 0, solid line) and ferroelectric (**p̅** ≠ 0, dashed line) phases.

## 4 From Infinite to Finite Dimensions

To assess the relevance of the mechanism identified in Sections 3.1–3.3 for real systems, it is important to clarify whether it persists at finite *d*. The central question is whether an annealed average over *r̂*_*ij*_ of a suitably renormalized dipolar two-body potential, *W*_*d*_, which incorporates many-body correlations encoded in higher-order terms of the virial expansion no longer negligible at finite *d*, generates an effective ferroelectric-like interaction. The many-body effects at finite-*d* can be accounted for through the renormalized dipolar potential defined within the optimized cluster expansion, introduced in Section 4.1. Section 4.2 then shows that its annealed average over *r̂*_*ij*_ indeed generates a ferroelectric-like dipolar interaction. In the present framework, the pair correlation function entering the DFT functional is related to the effective interaction through

63 $$V\left(r,{\mathrm{r̂}}_{ij},{\mathrm{d̂}}_{i},{\mathrm{d̂}}_{j}\right)=-\frac{1}{\beta }\log g^{\left(2\right)}\left(r,{\mathrm{r̂}}_{ij},{\mathrm{d̂}}_{i},{\mathrm{d̂}}_{j}\right)$$

up to a physically irrelevant additive constant. Consistently with Section 1, and as appropriate for the optimized cluster expansion adopted here, decompose *V* = *V*_0_ + *W*_*d*_, where *V*_0_ is the isotropic contribution of the reference system to *V* and *W*_*d*_ the effective two-body dipolar interaction, which is a functional of *w*_*p*_. Introducing the perturbation parameter λ as in eq 4,

64 $$V=V_{0}+\lambda W_{d}$$

Assuming that *W*_*d*_ = *W*_*d*_[*w*_*p*_] is analytic around *w*_*p*_ = 0, so that eq 17 can be generalized to functionals of *w*_*p*_, one obtains

65 $$\underset{R_{c}\to \infty }{\lim}F\left[\zeta \right]=-C_{W}^{d}p^{2}f_{d}\left(\epsilon \right)\frac{\Omega_{d}}{d}\int drr^{d-1}e^{-\beta V_{0}(r)}d{\mathrm{d̂}}_{i}d{\mathrm{d̂}}_{j}{\mathrm{d̂}}_{i}\cdot {\mathrm{d̂}}_{j}+\frac{1}{2}N\rho \Omega_{d}\int drr^{d-1}e^{-\beta V_{0}(r)}d{\mathrm{d̂}}_{i}d{\mathrm{d̂}}_{j}\zeta \left({\mathrm{d̂}}_{i}\right)\langle e^{-\beta W_{d}(r,{\mathrm{r̂}}_{ij},{\mathrm{d̂}}_{i},{\mathrm{d̂}}_{j})}-1\rangle_{{\mathrm{r̂}}_{ij}}\zeta \left({\mathrm{d̂}}_{j}\right)$$

The constant $C_{W}^{d}$ originates from the component of W_d_[*w*_*p*_] linear in *w*_*p*_. For $C_{W}^{d}>0$ , the first term on the right-hand side of eq 65, favors ferroelectric ordering. If $C_{W}^{d}$ is negative, it suppresses it.

In the Supporting Information, an analytical expression for the excess free energy at finite *d*, resulting from the second-order truncation of the virial expansion, is derived, and shown to be minimized by ferroelectric order for all finite *d* ≥ 3. However, truncating the virial expansion at second order is generally inadequate to accurately describe real systems. To address this limitation, the renormalized potential within the optimized cluster expansion is introduced below. While effectively incorporating many-body contributions, this construction still allows the free-energy functional F to retain the same formal structure as in the second-order virial truncation.

### 4.1 Effective Two-Body Dipolar Interaction as Perturbative Potential in the Optimized Cluster Expansion

By writing the interaction potential as a sum of a hard-core reference potential and a generic perturbative term, the optimized cluster expansion for classical fluids^16,37^ provides an approximate expression for *W*_*d*_, incorporating many-body effects arising from terms in the virial expansion beyond second order. Within this framework, *W*_*d*_ coincides, up to a sign and a factor β, with the so-called renormalized potential $C_{p}$ obtained from a resummation of the virial series. Its diagrammatic representation involves generalized chains in which density vertices are replaced by hypervertices, functionals of the pair correlation function of the reference system, *g*_0_(**r**_*i*_, **r**_*j*_). Physically, the renormalized potential describes an effective interaction in which the perturbation potential is screened by the local structure imposed on the fluid by the reference potential.^16^ Only the aspects relevant to the present treatment are summarized below. Further details can be found in Refs. 16, 37 . For simplicity, when referring to a generic case, the dipolar degrees of freedom are neglected and a potential depending only on the position vector is considered.

In the optimized cluster expansion the pair density entering the DFT expression of F , is given by the following exponential approximation^16,37^

66 $$\rho^{\left(2\right)}\left(r_{i},r_{j}\right)=\mathrm{\rho ̃}\left(r_{i}\right)g_{0}\left(r_{i},r_{j}\right)e^{C_{p}(r_{i},r_{j},\beta )}\mathrm{\rho ̃}\left(r_{j}\right)$$

Equation 66 is asymptotically correct^37^ in each of the four limits: (i) the low-density limit, where it recovers the virial expansion with truncation at second order;^16^ (ii) the high-density limit; (iii) the high-temperature limit or weak coupling; and (iv) the γ → 0 limit, where γ^–1^ is the range of the perturbation potential in the γ expansion, i.e., the long-range limit. In classical DFT, treating $C_{p}$ as a perturbative interaction, and defining ${\rho^{\left(2\right)}}_{\lambda }\left(r_{i},r_{j}\right)=\mathrm{\rho ̃}\left(r_{i}\right)g_{0}\left(r_{i},r_{j}\right)e^{\lambda C_{p}(r_{i},r_{j},\beta )}\mathrm{\rho ̃}\left(r_{j}\right)$ , following eq 66, substitution into eq 4 and integration over λ yield

67 $$F\left[\mathrm{\rho ̃}\right]=-\frac{1}{2}\int dr_{i}dr_{j}\mathrm{\rho ̃}\left(r_{i}\right)g_{0}\left(r_{i},r_{j}\right)\left[e^{C_{p}(r_{i},r_{j},\beta )}-1\right]\mathrm{\rho ̃}\left(r_{j}\right)$$

Defining $V_{0}\left(r_{i},r_{j}\right)=-\frac{1}{\beta }\log g_{0}\left(r_{i},r_{j}\right)$ , eq 67 can be recognized as the finite-*d* counterpart of the virial expansion truncated at second order exact in the limit *d* → ∞. In this representation, the bare perturbative interaction *w*_*p*_ is replaced by the renormalized potential −β^–1^ $C_{p}$ , while the factor $e^{-\beta v_{0}(r)}$ is replaced, through topological reduction,^16,37,54^ by *g*_0_(**r**_*i*_, **r**_*j*_).

The explicit expression for the potential $C_{p}$ follows from a diagrammatic resummation of the virial series. The Mayer function in eq 20, representing the elementary bond of the virial expansion, can be rephrased by replacing the factor $e^{-\beta v_{0}(r)}$ with *g*_0_(**r**_*i*_, **r**_*j*_) and expanding $f_{w_{p}}$ in powers of *w*_*p*_, yielding

68 $$\mathrm{f̃}\left(r_{i},r_{j}\right)=h_{0}\left(r_{i},r_{j}\right)+\left(1+h_{0}\left(r_{i},r_{j}\right)\right)\overset{\infty }{\underset{n=1}{\sum }}{\left(-1\right)}^{n}\beta^{n}{\left[w_{p}(r_{i},r_{j})\right]}^{n}$$

The tilde indicates that the Mayer function has been modified via topological reduction and *h*_0_(**r**_*i*_, **r**_*j*_) = *g*_0_(**r**_*i*_, **r**_*j*_) – 1. The bond *f̃* appearing in the virial diagrams can be decomposed into three contributions: a single *h*_0_ bond, any number of powers of *w*_*p*_ bonds, any number of powers of *w*_*p*_ bonds times *h*_0_ bond. The renormalized potential is obtained by resumming chains of *w*_*p*_ bonds in the virial series.^16,37^ In summary, one obtains

69 $$\rho^{2}C_{p}\left(r_{i},r_{j},\beta \right)=\rho^{2}\overset{\infty }{\underset{n=1}{\sum }}C_{p}^{\left(n\right)}\left(r_{i},r_{j},\beta \right)$$

Each term $C_{p}^{\left(n\right)}\left(r_{i},r_{j},\beta \right)$ corresponds to a convolution integral which, in Fourier space with conjugate variable **k**, reads

70 $$\rho^{2}{\mathrm{C̃}}_{p}^{\left(n\right)}\left(k,\beta \right)={\left(-1\right)}^{n}{\left(\beta \right)}^{n}{\left[{\mathrm{w̃}}_{p}(k){\mathrm{\Sigma ̃}}_{0}(k)\right]}^{n}{\mathrm{\Sigma ̃}}_{0}\left(k\right)$$

where ${\mathrm{C̃}}^{\left(n\right)}\left(k,\beta \right)$ , *w̃*_*p*_(**k**), and Σ̃_0_(**k**) denote the Fourier transforms respectively of $C_{p}^{\left(n\right)}\left(r_{i},r_{j}\right)$ , *w*_*p*_(**r**_*i*_, **r**_*j*_), and of the so-called hypervertex function, defined as

71 $$\Sigma_{0}\left(r_{i},r_{j}\right)=\rho \delta \left(r_{i},r_{j}\right)+\rho^{2}h_{0}\left(r_{i},r_{j}\right)$$

It generalizes the elementary vertex ρδ­(**r**_*i*_, **r**_*j*_) appearing in the standard diagrammatic virial expansion by incorporating the local structure of the reference system through *h*_0_. For example, for *n* = 1, the corresponding expression in real space reads

72 $$\rho^{2}C_{p}^{\left(1\right)}\left(r_{i},r_{j},\beta \right)=-\beta \int \int \Sigma_{0}\left(r_{i},r_{k}\right)w_{p}\left(r_{k},r_{l}\right)\Sigma_{0}\left(r_{l},r_{j}\right)dr_{k}dr_{l}$$

As follows from eqs 69–72, the renormalized potential can be read as an effective interaction in which the bare perturbation is screened by the local structure generated in the fluid by the reference potential.^16^

### 4.2 Annealed Averaging of the Renormalized Potential over Positional Disorder and Ferroelectricity in Finite Dimensions

Equation 67, which applies at finite *d*, can be recast in a form analogous to eq 22, which provides the exact expression for the free energy in the limit *d* → ∞, as

73 $$F\left[\mathrm{\rho ̃}\right]=-\frac{N\rho }{2}\Omega_{d}\int r^{d-1}drg_{0}\left(r\right)d{\mathrm{d̂}}_{i}d{\mathrm{d̂}}_{j}\zeta \left({\mathrm{d̂}}_{i}\right)\langle f_{C_{p}}\left(r,{\mathrm{r̂}}_{ij},{\mathrm{d̂}}_{i},{\mathrm{d̂}}_{j}\right)\rangle_{{\mathrm{r̂}}_{ij}}\zeta \left({\mathrm{d̂}}_{j}\right)$$

The modified Mayer function associated with the renormalized dipolar potential is

74 $$f_{C_{p}}\left(r,{\mathrm{r̂}}_{ij},{\mathrm{d̂}}_{i},{\mathrm{d̂}}_{j}\right)=e^{C_{p}}-1$$

Since $\langle C_{p}^{\left(1\right)}\rangle_{{\mathrm{r̂}}_{ij}}=0$ , as follows from eq 72 and the isotropy of Σ_0_, which preserves under convolution the angular dependence inherited from $v_{p}\left({\mathrm{r̂}}_{ij}\right)$ , the leading nonvanishing isotropic contributions arise at order *w*_*p*_^2^. Under the approximation that the dominant isotropic sector is given by the l=0 projection of *w*_*p*_^2^, weighted by Σ_0_, whose positivity is proven in Appendix B-(i), this contribution is non-negative. Therefore, by Jensen’s inequality, one can safely assume $\langle e^{C_{p}}-1\rangle_{{\mathrm{r̂}}_{ij}}\geq 0$ . Consequently, minimizing F in eq 73 is equivalent to maximizing $\langle f_{C_{p}}\rangle_{{\mathrm{r̂}}_{ij}}$ .

To evaluate $\langle f_{C_{p}}\rangle_{{\mathrm{r̂}}_{ij}}$ in eq 73, $e^{C_{p}}-1$ is expanded in powers of $C_{p}$ in real space. Using eq 69, one obtains

75 $$\langle e^{C_{p}}-1\rangle_{{\mathrm{r̂}}_{ij}}=\overset{\infty }{\underset{n=1}{\sum }}\overset{n}{\underset{l=1}{\sum }}\frac{1}{l!}\underset{\begin{array}{c} n_{1}+\cdot \cdot \cdot +n_{l}=n\\ n_{i}\geq 1 \end{array}}{\sum }\langle C_{p}^{\left(n_{1}\right)}\cdot \cdot \cdot C_{p}^{\left(n_{l}\right)}\rangle_{{\mathrm{r̂}}_{ij}}$$

It is convenient to decompose *w*_*p*_ as in the following. Let

76 $$w_{p}\left(r_{ij},{\mathrm{d̂}}_{i},{\mathrm{d̂}}_{j}\right)=v_{p}\left({\mathrm{r̂}}_{ij},{\mathrm{d̂}}_{i},{\mathrm{d̂}}_{j}\right)q_{p}\left(r\right)$$

with

$$v_{p}\left({\mathrm{r̂}}_{ij},{\mathrm{d̂}}_{i},{\mathrm{d̂}}_{j}\right)=d\left({\mathrm{d̂}}_{i}\cdot {\mathrm{r̂}}_{ij}\right)\left({\mathrm{d̂}}_{j}\cdot {\mathrm{r̂}}_{ij}\right)-{\mathrm{d̂}}_{i}\cdot {\mathrm{d̂}}_{j},,q_{p}\left(r\right)=-p^{2}{\left(\frac{l}{r}\right)}^{d}\theta \left(r-l\right)$$ 77

where *v*_*p*_ and *q*_*p*_ denote respectively the angular and isotropic radial contributions. eq 70 then becomes

78 $$\rho^{2}{\mathrm{C̃}}_{p}^{\left(n\right)}\left(k_{ij},{\mathrm{d̂}}_{i},{\mathrm{d̂}}_{j},\beta \right)={\left(\beta \right)}^{n}{\left[v_{p}({\mathrm{k̂}}_{ij},{\mathrm{d̂}}_{i},{\mathrm{d̂}}_{j})\right]}^{n}{\left[-{\mathrm{q̃}}_{p}(k){\mathrm{\Sigma ̃}}_{0}(k)\right]}^{n}{\mathrm{\Sigma ̃}}_{0}\left(k\right)$$

where **k**_*ij*_ = *kk̂*_*ij*_ and *q̃*_*p*_(*k*) denotes the l=2 spherical-Bessel transform of *q*_*p*_(*r*). Note that, as emphasized in eq 78, the Fourier transform

79 $${\mathrm{w̃}}_{p}\left(k_{ij},{\mathrm{d̂}}_{i},{\mathrm{d̂}}_{j}\right)=\int dr_{ij}e^{-ik_{ij}\cdot r_{ij}}w_{p}\left(r_{ij},{\mathrm{d̂}}_{i},{\mathrm{d̂}}_{j}\right)$$

preserves the tensorial angular structure of the dipolar interaction: the dependence on *r̂*_*ij*_ is mapped onto the corresponding dependence on *k̂*_*ij*_. This follows from the spherical-harmonic expansions of *v*_*p*_(*r̂*_*ij*_, *d̂*_*i*_, *d̂*_*j*_) and $e^{-ik_{ij}\cdot r_{ij}}$ . The integration over *r̂*_*ij*_ selects the same l=2 harmonic component appearing in the expansion of *v*_*p*_(*r̂*_*ij*_, *d̂*_*i*_, *d̂*_*j*_), so that the angular dependence remains unchanged, while the radial part is transformed through a spherical-Bessel transform with l=2 .

Define

80 $$C_{p}^{\left(n_{1},...,n_{l}\right)}\left(r_{ij},{\mathrm{d̂}}_{i},{\mathrm{d̂}}_{j}\right)\equiv \overset{l}{\underset{a=1}{\prod }}C_{p}^{\left(n_{a}\right)}\left(r_{ij},{\mathrm{d̂}}_{i},{\mathrm{d̂}}_{j}\right)$$

Expanding in spherical harmonics,

81 $$C_{p}^{\left(n_{1},...,n_{l}\right)}\left(r_{ij},{\mathrm{d̂}}_{i},{\mathrm{d̂}}_{j}\right)=\underset{l,\mu }{\sum }Q_{p,l}^{\left(n_{1},...,n_{l}\right)}\left(r\right)a_{l\mu }^{\left(n_{1},...,n_{l}\right)}\left({\mathrm{d̂}}_{i},{\mathrm{d̂}}_{j}\right)Y_{l\mu }\left({\mathrm{r̂}}_{ij}\right)$$

the Fourier transform acts diagonally on each angular sector:

82 $${\mathrm{C̃}}_{p}^{\left(n_{1},...,n_{l}\right)}\left(k_{ij},{\mathrm{d̂}}_{i},{\mathrm{d̂}}_{j}\right)=\underset{l,\mu }{\sum }{\mathrm{Q̃}}_{p,l}^{\left(n_{1},...,n_{l}\right)}\left(k\right)a_{l\mu }^{\left(n_{1},...,n_{l}\right)}\left({\mathrm{d̂}}_{i},{\mathrm{d̂}}_{j}\right)Y_{l\mu }\left({\mathrm{k̂}}_{ij}\right)$$

where

83 $${\mathrm{Q̃}}_{p,l}^{\left(n_{1},...,n_{l}\right)}\left(k\right)={\left(2\pi \right)}^{d/2}i^{-l}k^{1-\frac{d}{2}}\int_{0}^{\infty }drr^{d/2}Q_{p,l}^{\left(n_{1},...,n_{l}\right)}\left(r\right)J_{l+\frac{d}{2}-1}\left(kr\right)$$

*J*_*ν*_(*x*) is the Bessel function of the first kind of order *ν*. A completely analogous inverse relation holds in real space for $Q_{p,l}^{\left(n_{1},...,n_{l}\right)}\left(r\right)$ . Taking the angular averages $\langle \rangle_{{\mathrm{r̂}}_{ij}}$ and $\langle \rangle_{{\mathrm{k̂}}_{ij}}$ selects only the isotropic sector l=0 , yielding

$$\langle {\mathrm{C̃}}_{p}^{\left(n_{1},...,n_{l}\right)}\rangle_{{\mathrm{k̂}}_{ij}}={\mathrm{Q̃}}_{p,0}^{\left(n_{1},...,n_{l}\right)}\left(k\right)a_{00}^{\left(n_{1},...,n_{l}\right)},\langle C_{p}^{\left(n_{1},...,n_{l}\right)}\rangle_{{\mathrm{r̂}}_{ij}}=Q_{p,0}^{\left(n_{1},...,n_{l}\right)}\left(r\right)a_{00}^{\left(n_{1},...,n_{l}\right)}$$ 84

The radial integration entering eq 73 selects the component at *k* = 0

85 $$a_{00}^{\left(n_{1},...,n_{l}\right)}{\mathrm{Q̃}}_{p,0}^{\left(n_{1},...,n_{l}\right)}\left(0\right)=\Omega_{d}\int_{0}^{\infty }drr^{d-1}a_{00}^{\left(n_{1},...,n_{l}\right)}Q_{p,0}^{\left(n_{1},...,n_{l}\right)}\left(r\right)$$

Since w_p_(**r**) = v_p_(r̂)­q_p_(r), the radial coefficient $Q_{p,0}^{\left(n_{1},...,n_{l}\right)}$ ­(r) is obtained from successive convolutions involving only Σ_0_(r), and −q_p_(r). Because β > 0, Σ_0_(r) >0, see Appendix B-(i), and −q_p_(r) ≥ 0, every radial contribution entering these convolutions is positive. Therefore, $Q_{p,0}^{\left(n_{1},...,n_{l}\right)}\left(r\right)>0$ , which, by eq 85, implies ${\mathrm{Q̃}}_{p,0}^{\left(n_{1},...,n_{l}\right)}\left(0\right)>0$ .

The quantity $\langle {\mathrm{C̃}}_{p}^{\left(n_{1},...,n_{l}\right)}\left(0,{\mathrm{d̂}}_{i},{\mathrm{d̂}}_{j}\right)\rangle_{{\mathrm{k̂}}_{ij}}$ is the isotropic component of the zero-wavevector convolution product of *l* terms ${\mathrm{C̃}}_{p}^{\left(n_{\alpha }\right)}\left(q,{\mathrm{d̂}}_{i},{\mathrm{d̂}}_{j},\beta \right)$ , each given in eq 78. Each elementary factor ${\mathrm{C̃}}_{p}^{\left(n_{\alpha }\right)}$ entering the convolution has *q̂*-component ${\left[v_{p}(\mathrm{q̂},{\mathrm{d̂}}_{i},{\mathrm{d̂}}_{j})\right]}^{n_{\alpha }}$ . Expanding each factor in spherical harmonics,

86 $${\left[v_{p}(\mathrm{q̂},{\mathrm{d̂}}_{i},{\mathrm{d̂}}_{j})\right]}^{n_{\alpha }}=\underset{l,\mu }{\sum }a_{l\mu }^{\left(n_{\alpha }\right)}\left({\mathrm{d̂}}_{i},{\mathrm{d̂}}_{j}\right)Y_{l\mu }\left(\mathrm{q̂}\right)$$

the isotropic coefficient a_00_^(n_α_)^ is related to

87 $$\mu_{n_{\alpha }}=\langle {\left[v_{p}(\mathrm{q̂},{\mathrm{d̂}}_{i},{\mathrm{d̂}}_{j})\right]}^{n_{\alpha }}\rangle_{\mathrm{q̂}}$$

by $a_{00}^{\left(n_{\alpha }\right)}=\sqrt{\Omega_{d}}\mu_{n_{\alpha }}$ with $Y_{00}=1/\sqrt{\Omega_{d}}$ . Assuming that the dominant isotropic contribution is directly given by the product of l=0 projection of powers of *v*_*p*_, one obtains

88 $$\langle {\mathrm{C̃}}_{p}^{\left(n_{1},...,n_{l}\right)}\left(0,{\mathrm{d̂}}_{i},{\mathrm{d̂}}_{j}\right)\rangle_{{\mathrm{k̂}}_{ij}}=A_{n_{1},...,n_{l}}\overset{l}{\underset{\alpha =1}{\prod }}\mu_{n_{\alpha }}\left({\mathrm{d̂}}_{i}\cdot {\mathrm{d̂}}_{j}\right)$$

Here $A_{n_{1},...,n_{l}}={\left(\sqrt{\Omega_{d}}\right)}^{l}{\mathrm{Q̃}}_{p,0}^{\left(n_{1},...,n_{l}\right)}\left(0\right)$ .The positivity of $A_{n_{1},...,n_{l}}$ follows from the positivity of ${\mathrm{Q̃}}_{p,0}^{\left(n_{1},...,n_{l}\right)}\left(0\right)$ . Contributions to $\langle {\mathrm{C̃}}_{p}^{\left(n_{1},...,n_{l}\right)}\left(0,{\mathrm{d̂}}_{i},{\mathrm{d̂}}_{j}\right)\rangle_{{\mathrm{k̂}}_{ij}}$ generated by the coupling of higher-order harmonics in the expansion of powers of *v*_*p*_ are neglected here. The direct isotropic contribution is assumed to be dominant, thus considerably simplifying the analysis. As shown in Appendix B-(ii), for *d* ≥ 3,

89 $$\max\left[\mu_{n}\left({\mathrm{d̂}}_{i}\cdot {\mathrm{d̂}}_{j}\right)\right]=\mu_{n}\left(1\right)$$

which under eq 90 and implies that $\langle C_{p}^{\left(n_{1},...,n_{l}\right)}\left(r_{ij},{\mathrm{d̂}}_{i},{\mathrm{d̂}}_{j}\right)\rangle_{{\mathrm{r̂}}_{ij}}$ is also maximized for *d̂*_*i*_·*d̂*_*j*_ = 1.

Since $\langle C^{1}\rangle_{{\mathrm{r̂}}_{ij}}=0$ , the averaging over *r̂*_*ij*_ introduce, similarly to the case *d* → ∞, a screening of the dipolar interaction, making it shorter range.

The excess free energy in eq 65 is minimized at *d̂_i_* · *d̂_j_* = 1 provided the constant $C_{W}^{d}$ , which determines the sign of the reaction-field term, is positive. The sign of $C_{W}^{d}$ is fixed by the linear term in *w_p_* in the functional Taylor expansion of $e^{-\beta W_{d}}$ around *w*_*p*_ = 0, with $W_{d}=-\beta^{-1}C_{p}$ . Inspection of eq 70 shows that the only contribution to $C_{p}$ linear in *w*_*p*_ is $C_{p}^{\left(1\right)}$ . Taking the functional derivative of eq 72 with respect to *w*_*p*_ yields the product of two isotropic kernels Σ_0_, each depending on different spatial coordinates. The resulting contribution is therefore quadratic in the radial integral of Σ_0_ and hence positive. Consequently, *C*_W^d^_ is positive, so that both the intrinsic bulk and reaction-field contributions in eq 65 favor dipole alignment.

Beyond the exponential approximation eq 66, different representations of the pair density can be employed, raising the question of whether the ferroelectric-like character of the corresponding effective interaction is preserved. In the high-temperature approximation (HTA), corresponding to the first-order truncation of the lambda-expansion,^16^ the term $e^{-\beta W_{d}}-1$ in eq 65 is replaced by the bare dipolar interaction *w*_*p*_, and $C_{W}^{d}=1$ . The intrinsic bulk contribution in eq 65 thus vanishes. The reaction-field contribution, i.e., the first term in eq 65, instead remains ferroelectric-like. In the one-mode or random-phase approximation (RPA), the term $e^{-\beta W_{d}}-1$ in eq 65 is replaced by the square of the bare dipolar interaction. Consequently, the intrinsic bulk contribution to the excess free energy is minimized for (*d̂*_*i*_· *d̂*_*j*_)^2^ = 1, corresponding to quadrupolar (nematic) order. The reaction-field contribution remains instead always ferroelectric-like. The HPA, RPA, and exponential approximations, however, represent, in this order, increasingly accurate levels of description, as each includes a progressively larger subset of diagrams in the cluster expansion.

### 4.3 A Simplified Mean-Field Expression for the Excess Free Energy

Within mean-field classical DFT, replacing *W*_*d*_ by its orientationally averaged counterpart *W̅_d_* yields a finite contribution to the intrinsic bulk excess free energy. For the sake of simplicity, even at finite *d*, one may take *W̅*_*d*_ = *w̅*_*p*_, obtained by annealed averaging over r̂_*ij*_ of *w*_*p*_ instead of the renormalized potential introduced in Section 4.1. At finite *d*, this choice corresponds to assume exact the truncation of the virial expansion at second order. With the mean-field replacement *pd̂*_*i*__(j)_ = δ, one obtains in three-dimensional space

90 $${\mathrm{w̅}}_{p}^{\left(3\right)}\left(r,{\mathrm{d̂}}_{i},{\mathrm{d̂}}_{j}\right)=-\frac{1}{\beta }\log\int_{-1}^{1}d\theta_{ij}e^{-\beta p^{2}{\left(\frac{l}{r}\right)}^{3}\delta^{2}\left[1-3\theta_{ij}^{2}\right]}$$

where θ_*ij*_ = δ̂·*r̂*_*ij*_ ≡ cos α_*ij*_. Since the exponential term enhances configurations with $\theta_{ij}^{2}$ close to unity, the integral can be approximated by replacing the Boltzmann factor with its argument averaged over a suitable distribution of α_*ij*_. Choosing α_*ij*_ uniformly distributed over its period [0, 2π] yields the distribution $f\left(\theta_{ij}\right)=\frac{1}{\pi \sqrt{1-\theta_{ij}^{2}}}$ for θ_*ij*_ ∈ [−1,1], which enhances the contribution of configurations with $\theta_{ij}^{2}≃1$ . One may therefore approximate the effective potential by averaging *w*_*p*_ over a uniform distribution of the angle between δ̂ and *r̂*_*ij*_, as in Ref 13,

91 $${\mathrm{w̅}}_{p}^{\left(3\right)}\left(r,{\mathrm{d̂}}_{i},{\mathrm{d̂}}_{j}\right)≃p^{2}{\left(\frac{l}{r}\right)}^{3}\delta^{2}{\left\langle 1-3\theta_{ij}^{2}\right\rangle }_{\alpha_{ij}}$$

where $\langle \rangle_{\alpha_{ij}}$ denotes an average over a uniform distribution of α_*ij*_. Even though this approximate expression correctly captures the ferroelectric character of *w̅*_*p*_, it neglects the screening effect introduced by the annealed averaging, which should make *w̅*_*p*_ shorter-ranged than *w*_*p*_. In Ref 13, the emergence of ferroelectricity was related to positional disorder. In light of the results presented in this manuscript, the positional disorder in Ref. 13 is treated as annealed, tailored to the characteristics of a liquid.

## 5 Conclusion

The emergence of ferroelectricity in dipolar liquids, which has been confirmed by several numerical simulation studies^4–9^ and more recent experiments on liquid crystals^10–12^ still lacks a solid theoretical foundation. Studies conducted within a mean field approach^23,24,28,30^ remain inconclusive. In particular, they do not clarify whether the ferroelectric phase transition in dipolar liquids is a genuine bulk phenomenon or is instead driven by sample-shape dependent surface contribution to the free energy, which do not vanishes in the thermodynamic limit owing to the long-range nature of the dipolar interaction. This property underlies the so-called conditional convergence of the dipolar potential. It is noteworthy that numerical simulations performed using Ewald summation with conducting periodic boundary conditions^4–6,8,13^ , for which the surface contribution to the free energy vanishes, nevertheless exhibit behavior consistent with a ferroelectric phase transition. To shed light on this issue, one must move beyond the mean field framework and consider pair correlations between dipoles, as well as the associated mean force generating hindered dipolar rotation, in the spirit of Kirkwood treatment of the dielectric properties of polar liquids.^3^ In the Kirkwood approach focus is placed on the mean force acting between nearest-neighbor dipoles, thereby implicitly emphasizing the role of local structure in any analysis of the onset of ferroelectricity in dipolar liquids. Such an approach would not, however, distinguish between a solid and a liquid exhibiting similar local structures. In the present study, a mean reaction-field construction^2–4,25,38–40^ is introduced in order to isolate the intrinsic bulk contribution of dipolar interaction while making vanishing the surface term. The existence of a ferroelectric phase transition in dipolar liquids, intrinsic to the bulk, is then demonstrated. This finding is significant, as it establishes that the onset of a ferroelectric phase transition in dipolar liquids, in the thermodynamic limit, may directly affect intrinsic bulk properties, such as the liquid–liquid phase transition in supercooled water or the emergence of related thermodynamic anomalies.

As main result of the present study, the mean force responsible for a hindered dipolar rotation in dipolar liquids favoring dipolar alignment, and the resulting onset of a ferroelectric phase transition, is shown to emerge from the annealed averaging of the pair dipolar interaction over the relative orientation of the intermolecular separation vector between two particles. In the limit *d* → ∞, where the truncation of the virial expansion at the second order becomes exact, the dipolar interaction entering the annealed average coincides with the bare dipolar potential, and its annealed counterpart provides an exact description of the free energy. In finite dimensions *d* ≥ 3, this dipolar interaction is an effective two-body dipolar potential incorporating many-body effects, and corresponds to an approximation of the free energy within the optimized cluster expansion framework.^16,37^ Both the infinite- and finite-dimensional cases are analyzed within a classical DFT framework. Annealed averaging over the orientation of the intermolecular separation vector is well posed provided that, the system remains liquid in its positional degrees of freedom, independently of the dipolar configuration. In particular, the construction does not rely on any separation of characteristic time scales between dipolar and translational degrees of freedom. The onset of ferroelectricity in dipolar liquids thus emerges as an intrinsic property of the liquid state, rooted in the annealed disorder inherent to the liquid phase. Within this picture, unlike approaches based on short-range local structure, ferroelectric order arises not in spite of the liquid nature of the system, but because of it. This outcome parallels the absence of frustration in the Sherrington–Kirkpatrick model with annealed disorder, leading to ordered spin phases.^48,49^ This perspective raises further questions, such as whether quenched disorder also supports the onset of a ferroelectric phase transition, or how ferroelectric order, or its absence, interplays with the crystallization of dipolar liquids. The integration of the classical DFT with the replicated liquid theory^20^ would allow the present treatment to be extended to the case of dipolar glasses.

Within the present framework, an effective dipolar interaction emerges that is screened by the annealed positional disorder, shorter-ranged than the bare interaction, and ferroelectric-like. While screening induced by the free rotational motion of dipoles, appropriate to the paraelectric phase, has been extensively considered, see e.g., Refs 31,32 , commonly refereed as Keesom interaction,^33^ the screening originating from the annealed positional disorder characteristic of the liquid state has remained largely unexplored.

If the dipolar liquid is embedded in a nonconducting medium, both in numerical simulations and in real systems, any macroscopic polarization necessarily generates a depolarization field that depends on the sample shape and on the dielectric constant of the surrounding medium. As a consequence, in the ferroelectric phase, polarized domains can develop.^9,28,55–58^ The difficulty in stabilizing such a state in a liquid may, speculatively, account for the tendency of deeply supercooled water, possibly in a low-density polarized phase, to crystallize.

As a further result, the present study identifies the pair correlation function in the paraelectric phase as a diagnostic indicator of the propensity toward ferroelectric ordering, which can occur when the correlation function becomes positive. On this basis, a method is proposed to reconstruct the pair correlation function entering the DFT free-energy functional from the moments of the probability distribution of *d̂*_*i*_ · *d̂*_*j*_ in the paraelectric phase. The same issue has recently been addressed from a different perspective by integrating supervised machine learning into a classical DFT framework.^52^ Interestingly, the onset of ferroelectric ordering is also shown to leave a detectable signature on the radial pair correlation function marginalized over the dipolar degrees of freedom, namely in the radial distribution function *g* ^(2)^(*r*) measured in numerical simulations after integrating out dipolar variables.

The present study finally places the classical DFT developments of Ref. 13, which describes the interplay between ferroelectric and liquid–liquid phase transitions in dipolar liquids with reference to supercooled water, on more solid ground.

## Supplementary Material



## A Appendix

### Large-*d* Polarization, Scaling, and Free Energy

In this Appendix, the following results are derived:

(i) The ansatz ζ­(*d̂*) in eq 46 is shown to discriminate, in the limit *d* → ∞, between paraelectric and ferroelectric states.
(ii) The entropy loss associated with the breaking of orientational isotropy and the emergence of macroscopic polarization is shown to scale as *O*(*d*) in the large-*d* limit, and its explicit expression is obtained.
(iii) The large-*d* scaling of the molecular dipole moment in eq 23 and of the radial variable in eq 24 is shown to yield an excess free energy F of *O*(*d*).
(iv) The change in F induced by the onset of a polarized state is computed.
(v) The expression of ${\mathrm{f̅}}_{w_{p}}\left({\mathrm{d̂}}_{i}\cdot {\mathrm{d̂}}_{j},h\right)$ in eq 42 is established.

i Consider the ansatz
  A1
  $$\zeta \left(\mathrm{d̂}\right)=\frac{1}{Z_{d}\left(\delta \right)}e^{d\delta \cdot \mathrm{d̂}};,Z_{d}\left(\delta \right)=\int d\mathrm{d̂}e^{d\delta \cdot \mathrm{d̂}}$$
  In the following, it is shown that, for small *δ*, **δ** ≡ δδ̂ ∝ **p̅**, so that **δ** acts as a ferroelectric order parameter. Accordingly, *ζ*(*d̂*) describes a paraelectric state for **δ** = 0 and a ferroelectric state for **δ** ≠ 0.
  The molecular average dipole moment is defined as
  p̅≡p̅∫dd̂ζ(d̂)d̂
  A2
  Introducing the variable *u* = *d̂*·*δ̂*, the angular measure becomes
  A3
  $$d\mathrm{d̂}=\Omega_{d-1}{\left(1-u^{2}\right)}^{d-3/2}du;,u\in \left[-1,1\right]$$
  and eq 95 reads
  $$\mathrm{p̅}=\mathrm{p̅}\frac{\int_{-1}^{1}duu\Omega_{d-1}{\left(1-u^{2}\right)}^{d-3/2}e^{d\delta u}}{Z_{d}\left(\delta \right)}\mathrm{\delta ̂};,Z_{d}\left(\delta \right)=\int_{-1}^{1}du\Omega_{d-1}{\left(1-u^{2}\right)}^{d-3/2}e^{d\delta u}$$
  A4
  To prove eq 96, one considers the integral over the solid angle of a generic function *F*(*d̂*·δ̂),
  A5
  $$\int_{\Omega_{d}}d\mathrm{d̂}F\left(\mathrm{d̂}\cdot \mathrm{\delta ̂}\right)=2\int_{R^{d}}d^{d}x\delta \left(x^{2}-1\right)F\left(x\cdot \mathrm{\delta ̂}\right)$$
  where ***x*** = *xd̂*. The factor 2 in eq 98 follows from the relationship δ­(**x** ^2^ – 1) = δ­(*x −*1)/(2*x*). Choosing δ̂ as polar axis in the $R^{d}$ space and decomposing
  A6
  $$x=u\mathrm{\delta ̂}+x_{⊥}$$
  where $x_{⊥}\in R^{d-1}$ , **x**_⊥_ = ρ*n̂*, and **x**_⊥_ · δ̂ = 0, one obtains
  A7
  $$d^{d}x=dud^{d-1}x_{⊥}=du\rho^{d-2}d\rho d\mathrm{n̂}$$
  where d*n̂* is the angular measure in the space $R^{d-1}$ . Hence
  A8
  $$\int d\mathrm{d̂}F\left(\mathrm{d̂}\cdot \mathrm{\delta ̂}\right)=2\int_{-\infty }^{\infty }du\int_{0}^{\infty }\rho^{d-2}d\rho \int_{\Omega_{d-1}}d\mathrm{n̂}\delta \left(u^{2}+\rho^{2}-1\right)F\left(u\right)$$
  Using $\delta \left(u^{2}+\rho^{2}-1\right)=\delta \left(\rho -\sqrt{1-u^{2}}\right)/\left(2\rho \right)$ , one finds
  A9
  $$2\int_{0}^{\infty }\rho^{d-2}d\rho \int_{\Omega_{d-1}}d\mathrm{n̂}\delta \left(u^{2}+\rho^{2}-1\right)=\Omega_{d-1}{\left(1-u^{2}\right)}^{d-3/2};,u\in \left[-1,1\right]$$
  From eqs 101–102, eq 96 follows. eq 97 is then obtained by noting that *ζ*(*d̂*) is axially symmetric about δ̂, so that the angular integral selects only the component of *d̂* parallel to δ̂.
  In the limit *d* → ∞, the integrals in eq 97 can be evaluated by Laplace’s saddle-point method. To leading order in *d*, both reduce to integrals of the form
  A10
  $$I_{d}\left(\delta \right)=\int_{-1}^{1}due^{d\Phi_{\delta }(u)}g\left(u\right);,\Phi_{\delta }\left(u\right)=\delta u+\frac{1}{2}\log\left(1-u^{2}\right)$$
  Here *g*(*u*) = Ω_*d*–1_ *u* for the numerator in **p̅**, and *g*(*u*) = Ω_*d*–1_ for *Z*_*d*_. If Φ_δ_(*u*) has a unique global maximum at $u=\mathrm{u̅}\left(\delta \right)\in \left(-1,1\right):\Phi_{\delta }^{\prime }\left(\mathrm{u̅}\right)=0,\Phi_{\delta }^{\prime \prime }\left(\mathrm{u̅}\right)<0$ , then, by Laplace’s saddle-point method, one obtains to leading order
  A11
  $$I_{d}\left(\delta \right)≃g\left(\mathrm{u̅}\right)e^{d\Phi_{\delta }(\mathrm{u̅})}\sqrt{\frac{2\pi }{d\left|\Phi_{\delta }^{\prime \prime }\left(\mathrm{u̅}\right)\right|}}$$
  where primes denote derivatives with respect to *u*. In the present case
  A12
  $$\Phi_{\delta }^{\prime }\left(u\right)=\delta -\frac{u}{1-u^{2}}=0$$
  which yields
  A13
  $$\mathrm{u̅}\left(\delta \right)=\frac{\sqrt{1+4\delta^{2}}-1}{2\delta }$$
  Combining eqs 97 and 104, one finally obtains
  A14
  p̅=p̅u̅(δ)δ̂
  For small δ,
  A15
  $$\mathrm{p̅}=\mathrm{p̅}\delta +O\left(\delta^{3}\right)$$
ii The entropic difference between the paraelectric (***δ*** = 0) and ferroelectric (**δ** ≠ 0) states is computed in the large-*d* limit. The dipole orientational distributions in the two states are
  A16
  $$\zeta_{0}\left(\mathrm{d̂}\right)=\frac{1}{\Omega_{d}},,\zeta_{\delta }\left(\mathrm{d̂}\right)=\frac{1}{Z_{d}\left(\delta \right)}e^{d\delta \cdot \mathrm{d̂}}$$
  where *Z*_*d*_(**δ**) is defined in eq 94. The entropy functional is defined as
  A17
  S[ζ]=−N∫dd̂ζ(d̂)log⁡ζ(d̂)
  and the entropic difference reads
  A18
  $$\Delta S\left(\delta \right)\equiv S\left[\zeta_{\delta }\right]-S\left[\zeta_{0}\right]$$
  *S*[*ζ*_**0**_] is readily obtained,
  A19
  $$S\left[\zeta_{0}\right]=N\log\Omega_{d}$$
  while
  A20
  $$S\left[\zeta_{\delta }\right]=-Nd\delta \cdot \int d\mathrm{d̂}\zeta_{\delta }\left(\mathrm{d̂}\right)\mathrm{d̂}+N\log Z_{d}\left(\delta \right)$$
  From eqs 95 and 107 it follows
  A21
  $$S\left[\zeta_{\delta }\right]=-Nd\delta \mathrm{u̅}\left(\delta \right)+N\log Z_{d}\left(\delta \right)$$
  Expressed in terms of *u* = *d̂*·δ̂, *Z*_*d*_(***δ***), as given in eq 97, can be evaluated via Laplace’s saddle-point method, as in eq 104. From this it follows
  A22
  $$\log Z_{d}\left(\delta \right)=\log\Omega_{d-1}+d\Phi_{\delta }\left(\mathrm{u̅}\right)+o\left(d\right)$$
  where *u̅* = *u̅*(δ) is given in eq 106. For δ = 0, the saddle point is at *u* = 0, and therefore
  A23
  $$\log\Omega_{d}=\log Z_{d}\left(0\right)=\log\Omega_{d-1}+o\left(d\right)$$
  Combining eqs 111–116, one obtains
  A24
  $$\Delta S\left(\delta \right)=Nd\left[\Phi_{\delta }\left(\mathrm{u̅}\right)-\delta \mathrm{u̅}\right]+o\left(d\right)$$
  Using the explicit expression of Φ_δ_(*u̅*) in eq 103 yields
  A25
  $$\Delta S\left(\delta \right)=\frac{Nd}{2}\log\left(1-{\mathrm{u̅}}^{2}\left(\delta \right)\right)+o\left(d\right)$$
  which implies eq 48, since 0 ≤ *u̅* ^2^ < 1.
iii For convenience, the large-*d* scaling of *p* and *r* are reported again below,
  A26
  $$p^{2}=d{\mathrm{p̅}}^{2};,r=l\left(1+\frac{\log d+h}{d}\right),,h=O\left(1\right)$$
  In the large-*d* limit, the prefactor of *w*_*p*_ becomes
  $$p^{2}{\left(\frac{l}{r}\right)}^{d}=d{\mathrm{p̅}}^{2}\exp\left\{-d\log\left(1+\frac{\log d+h}{d}\right)\right\}\underset{d\to \infty }{\to }d{\mathrm{p̅}}^{2}e^{-(\log d+h)}={\mathrm{p̅}}^{2}e^{-h}$$
  A27
  which remains finite for *h* = *O*(1). Although eq 120 is strictly valid for *h* = *O*(1), it can be conveniently extended to *h* → +∞, since following eq 120 the rescaled dipolar interaction vanishes exponentially in this limit. The region
  A28
  $$l<r<l\left(1+\frac{\log d}{d}\right);,-\log d<h<0$$
  is, however, still accessible under the hard-core constraint *r*  > *l*. Writing
  A29
  h=−log⁡d+s,⁣s=O(1)
  one obtains,
  A30
  $$p^{2}{\left(\frac{l}{r}\right)}^{d}=d{\mathrm{p̅}}^{2}{\left(1+\frac{s}{d}\right)}^{-d}\underset{d\to \infty }{\to }d{\mathrm{p̅}}^{2}e^{-s}$$
  Since the angular factor entering *w*_*p*_ is not positive definite, the dipolar interaction can become unbounded, possibly inducing instability in the system. Therefore, the scaling in eq 119 corresponds to a well-defined large-*d* limit if the region in eq 121 is excluded. This is achieved by introducing, in the large-*d* limit, the effective core at $l_{\mathrm{eff}}=l\left(1+\frac{\log d}{d}\right)$ . More generally, any effective core enforcing *h* > −*h*_*c*_, with *h*_*c*_ = *O*(1), would merely change the finite upper bound of the dipolar interaction, without affecting its functional form in the large-*d* limit.
  From eq 119, one obtains
  A31
  $$dr=\frac{l}{d}dh;,r^{d-1}=l^{d-1}{\left(1+\frac{\log d+h}{d}\right)}^{d-1}\underset{d\to \infty }{\to }l^{d-1}de^{h}$$
  and finally eqs 26–29. These results show that, under the large-*d* scaling in eq 119, the excess free energy F scales as *O*(*d*), as the entropy does, provided the reduced density $\rho B_{2}^{\mathrm{HS}}$ is kept finite in the limit *d* → ∞.
iv In the following, the excess free-energy difference ΔF between the paraelectric (**δ** = 0) and ferroelectric (**δ** ≠ 0) states in the large-*d* limit is obtained. The excess free energy evaluated on the ansatz in eq 94 is decomposed as
  A32
  $$F\left[\zeta_{\delta }\right]=F\left[\zeta_{0}\right]+\Delta F\left(\rho ,\delta \right),,\Delta F\left(\rho ,\delta \right)=F\left[\zeta_{\delta }\right]-F\left[\zeta_{0}\right]$$
  From eqs 26–29, the excess free energy is
  A33
  $$F\left[\zeta \right]=-\frac{N\rho }{\beta }B_{2}^{\mathrm{HS}}d\int_{-\infty }^{\infty }dhe^{h}e^{-\beta v_{0}(h)}\int d{\mathrm{d̂}}_{i}d{\mathrm{d̂}}_{j}\zeta \left({\mathrm{d̂}}_{i}\right)\zeta \left({\mathrm{d̂}}_{j}\right){\mathrm{f̅}}_{w_{p}}\left({\mathrm{d̂}}_{i}\cdot {\mathrm{d̂}}_{j},h\right)$$
  with ${\mathrm{f̅}}_{w_{p}}\left({\mathrm{d̂}}_{i}\cdot {\mathrm{d̂}}_{j},h\right)$ given in eq 29. Using the ansatz in eq 94, introducing the variables
  A34
  $$u_{i}={\mathrm{d̂}}_{i}\cdot \mathrm{\delta ̂},,u_{j}={\mathrm{d̂}}_{j}\cdot \mathrm{\delta ̂}$$
  and using eq 96, in the limit *d* → ∞ one finds
  A35
  $$\zeta \left({\mathrm{d̂}}_{i}\right)\zeta \left({\mathrm{d̂}}_{j}\right)d{\mathrm{d̂}}_{i}d{\mathrm{d̂}}_{j}=\frac{\Omega_{d-1}^{2}}{Z_{d}^{2}\left(\delta \right)}e^{d\left[\delta (u_{i}+u_{j})+\frac{1}{2}\log(1-u_{i}^{2})+\frac{1}{2}\log(1-u_{j}^{2})\right]}du_{i}du_{j}$$
  As shown in point (v) below, the integral in eq 29 can be expressed in terms of Gaussian random variables, and the argument of the exponential in eq 29 remains *O*(1) under suitable condition depending on p̅ , in the large-*d* limit. Therefore, given eq 128, the integral in eq 126 can be evaluated for *d* → ∞ by the Laplace saddle-point method, following the same steps as in eqs 103–104, with ${\mathrm{f̅}}_{w_{p}}$ acting as *g*. The saddle-point condition reads
  A36
  $$u_{i}=u_{j}=\mathrm{u̅}$$
  with *u̅* in eq 106. At this point,
  A37
  $${\mathrm{d̂}}_{i}\cdot {\mathrm{d̂}}_{j}={\mathrm{u̅}}^{2}$$
  Indeed, upon decomposing
  $${\mathrm{d̂}}_{i(j)}=\mathrm{u̅}\mathrm{\delta ̂}+{\left[1-{\mathrm{u̅}}^{2}\right]}^{1/2}{ê}_{i(j)}$$
  A38
  with ***e***_*i*(*j*)_·δ̂ = 0, one obtains
  A39
  $${\mathrm{d̂}}_{i}\cdot {\mathrm{d̂}}_{j}={\mathrm{u̅}}^{2}+\left(1-{\mathrm{u̅}}^{2}\right)\left({ê}_{i}\cdot {ê}_{j}\right)$$
  As follows from eq 41, for *r̂*_*ij*_ uniformly distributed on the unit sphere in *d* dimensions, the quantity $\sqrt{d}\left({\mathrm{r̂}}_{ij}\cdot {\mathrm{d̂}}_{i}\right)$ converges in distribution, in the limit *d* → ∞, to a centered Gaussian variable with unit variance. In the subspace orthogonal to δ̂, the same holds for $\sqrt{d-1}\left({ê}_{i}\cdot {ê}_{j}\right)$ . Consequently, *ê*_*i*_·*ê*_*j*_ = *O*(*d* ^–1/2^), from which eq 130 follows.
  Equation 50 is thus established.
v ${\mathrm{f̅}}_{w_{p}}\left({\mathrm{d̂}}_{i}\cdot {\mathrm{d̂}}_{j},h\right)$ is defined in eq 29. Introduce the variables
  A40
  $$\theta_{i}={\mathrm{d̂}}_{i}\cdot {\mathrm{r̂}}_{ij},,\theta_{j}={\mathrm{d̂}}_{j}\cdot {\mathrm{r̂}}_{ij}$$
  To express the angular measure d*r̂*_*ij*_ in terms of θ_*i*_ and θ_*j*_, consider an orthonormal basis (*ê*_1_, *ê*_2_) of the plane spanned by *d̂*_*i*_ and *d̂*_*j*_,
  A41
  $${\mathrm{r̂}}_{ij}=\eta_{1}{ê}_{1}+\eta_{2}{ê}_{2}+r_{⊥}$$
  The variables *η*_1(2)_ parameterize the components of *r̂*_*ij*_ in the (*d̂*_*i*_, *d̂*_*j*_) plane, while $r_{⊥}\in R^{d-2}$ represents the component of *r̂*_*ij*_ orthogonal to this plane. Applying the same argument used to derive eq 96, now resolving the solid-angle element d*r̂*_*ij*_ in terms of the two variables *η*_1_ and *η*_2_ instead of a single variable *u*, one obtains
  A42
  $$d{\mathrm{r̂}}_{ij}=\Omega_{d-2}{\left(1-\eta_{1}^{2}-\eta_{2}^{2}\right)}^{d-4/2}d\eta_{1}d\eta_{2},,\eta_{1}^{2}+\eta_{2}^{2}\leq 1$$
  It remains to determine the relation between the differential elements d*η*_1_ d*η*_2_ and d*θ*_*i*_ d*θ*_*j*_. Since *d̂*_*i*_ and *d̂*_*j*_ lie in the (*ê*_1_, *ê*_2_) plane, the variables **t**_*ij*_ = (*θ*_*i*_, *θ*_*j*_) depend linearly on ***η*** = (*η*_1_, *η*_2_),
  A43
  $$t_{ij}=A\eta$$
  with
  A44
  $$A=\left(\begin{array}{ll} {\mathrm{d̂}}_{i}\cdot {ê}_{1} & {\mathrm{d̂}}_{i}\cdot {ê}_{2}\\ {\mathrm{d̂}}_{j}\cdot {ê}_{1} & {\mathrm{d̂}}_{j}\cdot {ê}_{2} \end{array}\right)$$
  It follows that
  A45
  $$\eta_{1}^{2}+\eta_{2}^{2}=\eta^{T}\eta =t_{ij}G^{-1}t_{ij}^{T}$$
  where **G** = *AA* ^*T*^ is the Gram matrix of *d̂*_*i*_, *d̂*_*j*_. Therefore, the integration domain becomes
  A46
  $$D=\left\{t_{ij}=\left(\theta_{i},\theta_{j}\right)\in R^{2}:t_{ij}G^{-1}t_{ij}^{T}\leq 1\right\}$$
  Moreover, for the linear transformation **t**_*ij*_ = *A* ***η***, the integration measure transforms as
  A47
  $$d\eta_{1}d\eta_{2}=\frac{dt_{ij}}{\left|\det A\right|}=\frac{dt_{ij}}{{\left(\det G\right)}^{1/2}}$$
  where the identity det**G** = det­(*AA* ^*T*^) = (det *A*)^2^ has been exploited, and d**t**_*ij*_ = d*θ*_*i*_ d*θ*_*j*_. eq 40 is thus obtained. Introducing then the variable
  A48
  $${\mathrm{t̃}}_{ij}=\sqrt{d}t_{ij}$$
  one has
  A49
  $${\left(1-t_{ij}G^{-1}t_{ij}^{T}\right)}^{d-4/2}=e^{\frac{d-4}{2}\log\left(1-\frac{{\mathrm{t̃}}_{ij}G^{-1}{\mathrm{t̃}}_{ij}^{T}}{d}\right)}\underset{d\to \infty }{\to }e^{-\frac{1}{2}{\mathrm{t̃}}_{ij}G^{-1}{\mathrm{t̃}}_{ij}^{T}}$$
  Moreover, using $\Omega_{d}=\frac{2\pi^{d/2}}{\Gamma \left(d/2\right)}$ , where Γ­(*x*) denotes the Gamma function satisfying Γ­(*x* + 1) = *x*Γ­(*x*), one obtains
  A50
  $$\frac{\Omega_{d-2}}{\Omega_{d}}=\frac{d-2}{2\pi }$$
  Since
  A51
  $$d{\mathrm{t̃}}_{ij}=ddt_{ij}$$
  Equation 41 is thus retrieved. The large-*d* scaling in eq 119 finally leads to eq 42. Equation 44 shows that *f̅*(*r*, *r̂*_*ij*_, *d̂*_*i*_, *d̂*_*j*_) can be rewritten in terms of the moment-generating function *M*_*Z̃*_(*t*) of the random variable *Z̃* = *θ̃*_*i*_ *θ̃*_*j*_ – *d̂*_*i*_·*d̂*_*j*_:
  A52
  $$M_{\mathrm{Z̃}}\left(t\right)=\left\langle e^{t({\mathrm{\theta ̃}}_{i}{\mathrm{\theta ̃}}_{j}-{\mathrm{d̂}}_{i}\cdot {\mathrm{d̂}}_{j})}\right\rangle =e^{-t{\mathrm{d̂}}_{i}\cdot {\mathrm{d̂}}_{j}}\left\langle e^{t{\mathrm{\theta ̃}}_{i}{\mathrm{\theta ̃}}_{j}}\right\rangle$$
  Since (*θ̃*_*i*_, *θ̃*_*j*_) is distributed as a centered bivariate Gaussian with covariance matrix **G**, one can rewrite
  $$-\frac{1}{2}{\mathrm{t̃}}_{ij}^{T}G^{-1}{\mathrm{t̃}}_{ij}+t{\mathrm{\theta ̃}}_{i}{\mathrm{\theta ̃}}_{j}=-\frac{1}{2}{\mathrm{t̃}}_{ij}^{T}\left(G^{-1}-tĨ\right){\mathrm{t̃}}_{ij}$$
  where **ĩ** is the symmetric matrix with vanishing diagonal entries and unit off-diagonal entries. Performing the Gaussian integral then gives,
  A53
  $$\left\langle e^{t{\mathrm{\theta ̃}}_{i}{\mathrm{\theta ̃}}_{j}}\right\rangle =\frac{1}{\sqrt{\det G\det\left(G^{-1}-tĨ\right)}}$$
  Using
  A54
  $$\det G\det\left(G^{-1}-tĨ\right)=1-2t{\mathrm{d̂}}_{i}\cdot {\mathrm{d̂}}_{j}-t^{2}\left(1-q^{2}\right)$$
  one finally obtains eq 45.

## B Appendix

### Properties of Σ_0_ and *μ*_*n*_ at Finite Dimension

This Appendix establishes the following results:

(i) The positivity of the hypervertex function Σ_0_(*r*) entering $C_{p}^{\left(n\right)}$ for dipolar interaction.
(ii) The quantities μ_*n*_ defined in eq 89 are maximal when *d̂*_*i*_· *d̂*_*j*_ = 1.

i It is convenient to rewrite the hypervertex function in terms of the pair correlation function *g*_0_(**r**_*i*_, **r**_*j*_) rather than *h*_0_(**r**_*i*_, **r**_*j*_). Equation 71 then becomes
  B1
  $$\Sigma_{0}\left(r_{i},r_{j}\right)=\rho \delta \left(r_{i}-r_{j}\right)-\rho^{2}+\rho^{2}g_{0}\left(|r_{i}-r_{j}|\right)$$
  Consider the generic $C_{p}^{\left(n\right)}$ obtained through generalization of eq 72,
  $$\rho^{2}C_{p}^{\left(n\right)}=-\beta \int dr_{1}\cdot \cdot \cdot dr_{n+1}\left[\rho \delta \left(r_{i}-r_{1}\right)-\rho^{2}+\rho^{2}g_{0}\left(|r_{i}-r_{1}|\right)\right]w_{p}\left(r_{1}-r_{2},{\mathrm{d̂}}_{1},{\mathrm{d̂}}_{2}\right)\Sigma_{0}\left(r_{2},r_{3}\right)\cdot \cdot \cdot w_{p}\left(r_{n}-r_{n+1},{\mathrm{d̂}}_{n},{\mathrm{d̂}}_{n+1}\right)\Sigma_{0}\left(r_{n+1},r_{j}\right)$$
  B2
  If the term −ρ^2^ is selected from the first hypervertex Σ_0_(**r**_*i*_, **r**_1_) = [ρ*δ*(**r**_*i*_ – **r**_1_) – ρ^2^ + ρ^2^ *g*_0_(|**r**_*i*_ – **r**_1_|)], the integration over **r**_1_ factorizes and produces a term proportional to
  B3
  $$\int dr_{1}w_{p}\left(r_{1}-r_{2},{\mathrm{d̂}}_{1},{\mathrm{d̂}}_{2}\right)=0$$
  because $\langle v_{p}\left({\mathrm{r̂}}_{ij},{\mathrm{d̂}}_{i},{\mathrm{d̂}}_{j}\right)\rangle_{{\mathrm{r̂}}_{ij}}=0$ . The same holds whenever the term −ρ^2^ is selected from any hypervertex Σ_0_(**r**_*m*–1_,**r**_*m*_) in the convolution chain: the integration over **r**_*m*–1_ factorizes and yields an integral proportional to
  B4
  $$\int dr_{m-1}w_{p}\left(r_{m-1}-r_{m},{\mathrm{d̂}}_{m-1},{\mathrm{d̂}}_{m}\right)$$
  which again vanishes by eq 150. Therefore, every contribution containing the constant term −ρ^2^ vanishes identically at any order in $C_{p}^{\left(n\right)}$ , ∀ *n*. The hypervertex can thus be replaced by the effective isotropic function
  B5
  $${\mathrm{\Sigma ̃}}_{0}\left(r\right)=\rho \frac{\delta \left(r\right)}{\Omega_{d}r^{d-1}}+\rho^{2}g_{0}\left(r\right)$$
  where the first term is the radial representation of the *d*-dimensional Dirac delta. In the nontrivial case where *g*_0_(*r*) is not identically zero, one has *g*_0_(*r*) > 0 on a finite interval, and consequently Σ̃_0_(*r*) is positive.
ii The quantities *μ*_*n*_ in eq 89 are shown to be maximized when *d̂*_*i*_ · *d̂*_*j*_ = 1. Note that in the following the integration variable has been changed from *q̂* in eq 89 to *r̂*_*ij*_ purely for notational convenience, since it is a dummy variable. Defining
  B6
  $$v_{p}\left({\mathrm{r̂}}_{ij},{\mathrm{d̂}}_{i},{\mathrm{d̂}}_{j}\right)={\mathrm{r̂}}_{ij}^{T}A\left({\mathrm{d̂}}_{i},{\mathrm{d̂}}_{j}\right){\mathrm{r̂}}_{ij}$$
  with *A*(*d̂*_*i*_, *d̂*_*j*_) the real symmetric *d* × *d* matrix
  B7
  $$A\left({\mathrm{d̂}}_{i},{\mathrm{d̂}}_{j}\right)=\frac{d}{2}\left({\mathrm{d̂}}_{i}{\mathrm{d̂}}_{j}^{T}+{\mathrm{d̂}}_{j}{\mathrm{d̂}}_{i}^{T}\right)-{\mathrm{d̂}}_{i}\cdot {\mathrm{d̂}}_{j}I$$
  one can write, up to the positive normalization factor $\frac{1}{\Omega_{d}}$ ,
  B8
  $$\mu_{n}=\int d{\mathrm{r̂}}_{ij}{\left({\mathrm{r̂}}_{ij}^{T}A({\mathrm{d̂}}_{i},{\mathrm{d̂}}_{j}){\mathrm{r̂}}_{ij}\right)}^{n}$$
  Consider the generalized binomial expansion
  B9
  $${\left(1-x\right)}^{-d/2}=\overset{\infty }{\underset{n=0}{\sum }}\frac{(d/2{)}_{n}}{n!}x^{n}$$
  Here
  B10
  $$\frac{(d/2{)}_{n}}{n!}=\frac{\Gamma \left(d/2+n\right)}{\Gamma \left(d/2\right)\Gamma \left(n+1\right)}$$
  It is then convenient to introduce the auxiliary generating function
  B11
  $$H_{{\mathrm{d̂}}_{i}\cdot {\mathrm{d̂}}_{j}}\left(z\right)=\overset{\infty }{\underset{n=0}{\sum }}\frac{(d/2{)}_{n}}{n!}\mu_{n}z^{n}$$
  The quantities *μ*_*n*_ are therefore directly encoded in the Taylor coefficients of $H_{{\mathrm{d̂}}_{i}\cdot {\mathrm{d̂}}_{j}}\left(z\right)$ , up to the positive factors (*d*/2)_*n*_/*n*!. Using eq 155, exchanging the order of summation and integration, and applying eq 156 with *x* = *zv*_*p*_(*r̂*_*ij*_, *d̂*_*i*_, *d̂*_*j*_), one obtains
  B12
  $$H_{{\mathrm{d̂}}_{i}\cdot {\mathrm{d̂}}_{j}}\left(z\right)=\int d{\mathrm{r̂}}_{ij}{\left[1-z{\mathrm{r̂}}_{ij}^{T}A({\mathrm{d̂}}_{i},{\mathrm{d̂}}_{j}){\mathrm{r̂}}_{ij}\right]}^{-d/2}$$
  The function $H_{{\mathrm{d̂}}_{i}\cdot {\mathrm{d̂}}_{j}}\left(z\right)$ is introduced because studying the sign and monotonicity of *μ*_*n*_ is equivalent to studying its Taylor coefficients.
  To evaluate $H_{{\mathrm{d̂}}_{i}\cdot {\mathrm{d̂}}_{j}}\left(z\right)$ , it is convenient to work in the eigenbasis of *A*(*d̂*_*i*_, *d̂*_*j*_). Since it is a real symmetric matrix, it can be diagonalized by an orthogonal transformation. In its eigenbasis,
  B13
  $${\mathrm{r̂}}_{ij}^{T}A\left({\mathrm{d̂}}_{i},{\mathrm{d̂}}_{j}\right){\mathrm{r̂}}_{ij}=\overset{d}{\underset{\alpha =1}{\sum }}\lambda_{\alpha }r_{ij,\alpha }^{2}$$
  where λ_α_ are the eigenvalues of *A*(*d̂*_*i*_, *d̂*_*j*_), α = 1, ···, *d* labels the corresponding orthonormal eigenvectors ***e***_α_, and *r*_*ij,*α_ = *r̂*_*ij*_ · ***e***_*α*_. The eigenvalues of *A*(*d̂*_*i*_, *d̂*_*j*_) are
  B14
  $$\lambda_{\pm }=\frac{\left(d-2\right)\left({\mathrm{d̂}}_{i}\cdot {\mathrm{d̂}}_{j}\right)\pm d}{2},,\lambda_{0}=-\left({\mathrm{d̂}}_{i}\cdot {\mathrm{d̂}}_{j}\right)$$
  where *λ*_0_ has multiplicity *d* – 2. To derive eq 161, one can choose an orthonormal basis
  B15
  $${\mathrm{d̂}}_{i}=e_{1},,{\mathrm{d̂}}_{j}=\left({\mathrm{d̂}}_{i}\cdot {\mathrm{d̂}}_{j}\right)e_{1}+{\left[1-{\left({\mathrm{d̂}}_{i}\cdot {\mathrm{d̂}}_{j}\right)}^{2}\right]}^{1/2}e_{2}$$
  On the plane spanned by ***e***_1_ and ***e***_2_, the restriction of *A*(*d̂*_*i*_, *d̂*_*j*_) takes the form
  B16
  $$A\left({\mathrm{d̂}}_{i},{\mathrm{d̂}}_{j}\right)=\left(\begin{array}{ll} \left(d-1\right)\left({\mathrm{d̂}}_{i}\cdot {\mathrm{d̂}}_{j}\right) & \frac{d}{2}{\left[1-{\left({\mathrm{d̂}}_{i}\cdot {\mathrm{d̂}}_{j}\right)}^{2}\right]}^{1/2}\\ \frac{d}{2}{\left[1-{\left({\mathrm{d̂}}_{i}\cdot {\mathrm{d̂}}_{j}\right)}^{2}\right]}^{1/2} & -\left({\mathrm{d̂}}_{i}\cdot {\mathrm{d̂}}_{j}\right) \end{array}\right)$$
  On the orthogonal complement, one has
  B17
  $$A_{⊥}\left({\mathrm{d̂}}_{i},{\mathrm{d̂}}_{j}\right)=-\left({\mathrm{d̂}}_{i}\cdot {\mathrm{d̂}}_{j}\right)I$$
  so that λ_0_ = −(*d̂*_*i*_ · *d̂*_*j*_) is an eigenvalue with multiplicity *d* – 2. The remaining two eigenvalues are those of the restricted matrix $A\left({\mathrm{d̂}}_{i},{\mathrm{d̂}}_{j}\right)$ . In the diagonal basis of *A*(*d̂*_*i*_, *d̂*_*j*_),
  B18
  $$H_{{\mathrm{d̂}}_{i}\cdot {\mathrm{d̂}}_{j}}\left(z\right)=\int d{\mathrm{r̂}}_{ij}{\left(1-z\sum_{\alpha =1}^{d}\lambda_{\alpha }r_{ij,\alpha }^{2}\right)}^{-d/2}$$
  The uniform measure on the unit sphere can equivalently be generated from Gaussian variables as detailed in the following. Let $g=gĝ\in R^{d}$ be a vector distributed according to the normalized Gaussian measure
  B19
  $$dP\left(g\right)=\frac{dge^{-g^{2}/2}}{\int_{R^{d}}dge^{-g^{2}/2}}$$
  Then the unit vector $ĝ=\frac{g}{\left|g\right|}$ is uniformly distributed on the *d*-dimensional unit sphere. Therefore,
  B20
  $$H_{{\mathrm{d̂}}_{i}\cdot {\mathrm{d̂}}_{j}}\left(z\right)=\frac{\int_{R^{d}}dge^{-g^{2}/2}{\left(1-z\sum_{\alpha =1}^{d}\lambda_{\alpha }\frac{g_{\alpha }^{2}}{g^{2}}\right)}^{-d/2}}{\int_{R^{d}}dge^{-g^{2}/2}}$$
  Using spherical coordinates with d**g** = *g* ^*d*–1^ d*g* d*ĝ* and *g*_α_/*g* = *ĝ*_α_, the radial integral factorizes both in the numerator and in the denominator and cancels, yielding
  B21
  $$H_{{\mathrm{d̂}}_{i}\cdot {\mathrm{d̂}}_{j}}\left(z\right)=\frac{1}{\Omega_{d}}\int dĝ{\left(1-z\sum_{\alpha =1}^{d}\lambda_{\alpha }{ĝ}_{\alpha }^{2}\right)}^{-d/2}$$
  Define
  B22
  $$I\left(z\right)=\int_{R^{d}}dge^{-\frac{1}{2}\sum_{\alpha =1}^{d}(1-z\lambda_{\alpha })g_{\alpha }^{2}}$$
  Using spherical coordinates, it transforms into
  B23
  $$I\left(z\right)=\int dĝ\int_{0}^{\infty }dgg^{d-1}e^{-g^{2}/2\left(1-z\sum_{\alpha =1}^{d}\lambda_{\alpha }{ĝ}_{\alpha }^{2}\right)}$$
  Since
  B24
  $$\int_{0}^{\infty }dgg^{d-1}e^{-\frac{1}{2}ag^{2}}=2^{\frac{d}{2}-1}\Gamma \left(\frac{d}{2}\right)a^{-d/2}$$
  it follows that
  B25
  $$I\left(z\right)=2^{\frac{d}{2}-1}\Gamma \left(\frac{d}{2}\right)\int dĝ{\left(1-z\sum_{\alpha =1}^{d}\lambda_{\alpha }{ĝ}_{\alpha }^{2}\right)}^{-d/2}$$
  Computing *I*(*z*) at *z* = 0, one obtains
  B26
  $$H_{{\mathrm{d̂}}_{i}\cdot {\mathrm{d̂}}_{j}}\left(z\right)=\frac{I\left(z\right)}{I\left(0\right)}$$
  On the other hand,
  B27
  $$I\left(z\right)=\overset{d}{\underset{\alpha =1}{\prod }}\int_{-\infty }^{\infty }dg_{\alpha }e^{-\frac{1}{2}(1-z\lambda_{\alpha })g_{\alpha }^{2}}$$
  Using
  B28
  $$\int_{-\infty }^{\infty }dge^{-\frac{1}{2}ag^{2}}=\sqrt{\frac{2\pi }{a}}$$
  thus one finally obtains
  B29
  $$H_{{\mathrm{d̂}}_{i}\cdot {\mathrm{d̂}}_{j}}\left(z\right)=\overset{d}{\underset{\alpha =1}{\prod }}{\left(1-z\lambda_{\alpha }\right)}^{-1/2}$$
  With *q* = *d̂*_*i*_· *d̂*_*j*_, from eq 176, and expanding −log­(1 – *x*) in powers of *x*, one obtains
  B30
  $$\log H_{q}\left(z\right)=\overset{\infty }{\underset{m=1}{\sum }}\frac{p_{m}\left(q\right)}{2m}z^{m};,p_{m}\left(q\right)=\overset{d}{\underset{\alpha =1}{\sum }}\lambda_{\alpha }^{m}$$
  Using the spectrum of *A*(*d̂*_*i*_, *d̂*_*j*_), see eq 161,
  B31
  $$p_{m}\left(q\right)={\left[\frac{d-2}{2}q+\frac{d}{2}\right]}^{m}+{\left[\frac{d-2}{2}q-\frac{d}{2}\right]}^{m}+\left(d-2\right){\left(-q\right)}^{m}$$
  Since *A*(*d̂*_*i*_, *d̂*_*j*_) is traceless, *p*_1_(*q*) = 0. As follows immediately from the explicit form of the eigenvalues, *p*_*m*_(−*q*) = (−1)^*m*^ *p*_*m*_(*q*). It is therefore sufficient to analyze the sign and monotonicity properties for *q* > 0. For *d* ≥ 3 and 0 < *q* ≤ 1, one has
  B32
  $$p_{m}\left(q\right)>0,,m\geq 2$$
  For even *m*, this is immediate. For odd *m* ≥ 3, using the binomial expansion, one obtains
  B33
  (d2+d−22q)m−(d2−d−22q)m=2∑lodd(ml)(d2)m−l(d−22q)l
  All terms in the sum are positive. Keeping only the first term gives $m\left(d-2\right){\left(\frac{d}{2}\right)}^{m-1}q$ . Since *m* ≥ 3, *d* ≥ 3, and 0 < *q* ≤ 1, one has $m{\left(\frac{d}{2}\right)}^{m-1}q>q^{m}$ . Therefore, eq 179 follows. Moreover, by the same argument, one obtains
  B34
  $$\frac{dp_{m}\left(q\right)}{dq}>0,,m\geq 2,,q>0$$
  Thus, all nonzero Taylor coefficients of log *H*_*q*_(*z*) and of ∂_*q*_ log *H*_*q*_(*z*) are positive for *q* > 0. Since
  B35
  $$H_{q}\left(z\right)=\exp\left[\log H_{q}\left(z\right)\right];,\partial_{q}H_{q}\left(z\right)=H_{q}\left(z\right)\partial_{q}\log H_{q}\left(z\right)$$
  the Taylor coefficients of *H*_*q*_(*z*) and of ∂_*q*_ *H*_*q*_(*z*) are themselves positive. Comparing with eq 158 and considering that (*d*/2)_*n*_/*n*! > 0, it follows
  B36
  $$\mu_{n}\left(q\right)>0,,\frac{d\mu_{n}\left(q\right)}{dq}>0$$
  for *n* ≥ 2, *d* ≥ 3, and *q* > 0. Moreover, since *p*_*m*_(−*q*) = (−1)^*m*^ *p*_*m*_(*q*), the moments inherit the same parity structure: *μ*_*n*_ is even for even *n* and odd for odd *n*. Furthermore, for even *n*, ∂_*q*_ *μ*_*n*_ is odd, whereas for odd *n* it is even. Therefore, eq 183 implies that, for even *n*, *μ*_*n*_ increases with |*q*|, while for odd *n* it is monotonically increasing on the whole interval [−1, 1]. In particular,
  B37
  $$\mu_{n}\left(q\right)\leq \mu_{n}\left(1\right)$$
  so that ∀n , *μ*_*n*_ are maximal for *q* = 1.

## Notes

Second virial coefficient of dipolar interactions in finite dimension (PDF)
