# Supplementary material for: Ferroelectricity in Dipolar Liquids: The Role of Annealed Positional Disorder
Source: J Phys Chem B. 2026 Jun 29;130(27):6933–54. doi: 10.1021/acs.jpcb.5c08120 (PMC13359381; doi:10.1021/acs.jpcb.5c08120)
Supplement: Supplementary file 1 [file jp5c08120_si_002.pdf]

Ferroelectricity in dipolar liquids: the role of annealed positional disorder. *Supplementary Material*

Maria Grazia Izzo*

Department of Molecular Sciences and Nanosystems, Ca' Foscari University of Venice, Via Torino 155, 30172 Venezia Mestre, Italy

E-mail: mariagrazia.izzo@unive.it

Second Virial Coefficient of Dipolar Interactions in Finite Dimension

In finite d, one obtains

$$\langle f_{w_p}(r, \hat{r}_{ij}, \hat{d}_i, \hat{d}_j)\rangle_{\hat{r}_{ij}} = M_{\tilde{Z}}(t) - 1, \tag{1}$$

where $M_{\tilde{Z}}(t)$ is the moment-generating function of $\tilde{Z}$, defined in Eq. 43 in the main text, for the probability distribution appearing on the left-hand side of Eq. 41 in the main text. For finite-d, $t = \beta p^2 \left(\frac{l}{r}\right)^d$. Following the steps detailed in point (i) below, one obtains

$$M_{\tilde{Z}}(t) = e^{-t\hat{d}_i\cdot\hat{d}_j} 4\pi \frac{\Omega_{d-2}}{\Omega_d} \int_0^1 du\ (1-u)^{\frac{d-4}{2}} e^{\hat{d}_i\cdot\hat{d}_j t \frac{d}{2} u} I_0\big(t\frac{d}{2}u\big), \tag{2}$$

where $I_0(x) = \frac{1}{2\pi}\int_0^{2\pi} e^{x\cos\phi} d\phi$ is the modified Bessel function of order zero. The integral in Eq. 2 converges for all $d \geq 3$ and finite t. Jensen's inequality moreover implies $M_{\tilde{Z}}(t) \geq 1$, $\forall t$. Consequently, the contribution to the excess free energy arising from the second-order truncation of the virial expansion, Eq. 22 in the main text, is minimized when $M_{\tilde{Z}}(t)$ is maximal. As shown in point (ii) below, $M_{\tilde{Z}}(t, \hat{d}_i \cdot \hat{d}_j)$ in Eq. 2 attains its maximum at $\hat{d}_i \cdot \hat{d}_j = 1$.

Below, the following results are derived:

(i) An analytical expression for $\langle f_{w_p}(r, \hat{r}_{ij}, \hat{d}_i, \hat{d}_j)\rangle_{\hat{r}_{ij}}$ at finite d.

(ii) The second-order virial contribution to the free energy at finite $d \geq 3$ attains its minimum for $\hat{d}_i \cdot \hat{d}_j = 1$.

(i) The moment-generating function of $\tilde{Z}$ appearing in Eq. 1, associated with the probability distribution in the left-hand side of Eq. 41 in the main text, is

$$M_{\tilde{Z}}(t) = e^{-t\hat{d}_i\cdot\hat{d}_j}\frac{\Omega_{d-2}}{\Omega_d}\int d\boldsymbol{t}_{ij}\,\left(1 - \boldsymbol{t}_{ij}^{\mathrm{T}}\mathbf{G}^{-1}\boldsymbol{t}_{ij}\right)^{\frac{d-4}{2}} e^{td\,\theta_i\theta_j}. \tag{3}$$

Being $\mathbf{G}^{-1}$ the Gramm matrix with entries $\hat{d}_i \cdot \hat{d}_j$, explicitly,

$$\boldsymbol{t}_{ij}^{\mathrm{T}}\mathbf{G}^{-1}\boldsymbol{t}_{ij} = \frac{\theta_i^2 + \theta_j^2 - 2(\hat{d}_i\cdot\hat{d}_j)\theta_i\theta_j}{1 - (\hat{d}_i\cdot\hat{d}_j)^2}. \tag{4}$$

With the change of variables $(\theta_i, \theta_j) \to (\eta, \zeta)$, where

$$\eta = \frac{\theta_i + \theta_j}{\sqrt{2(1 + \hat{d}_i\cdot\hat{d}_j)}}, \qquad \zeta = \frac{\theta_i - \theta_j}{\sqrt{2(1 - \hat{d}_i\cdot\hat{d}_j)}}, \tag{5}$$

the matrix $\boldsymbol{G}^{-1}$ becomes diagonal. The integration measure transforms as

$$d\eta\, d\zeta = \frac{d\theta_i\, d\theta_j}{\sqrt{1 - (\hat{d}_i\cdot\hat{d}_j)^2}}. \tag{6}$$

Furthermore,

$$\boldsymbol{t}_{ij}^{\mathrm{T}}\mathbf{G}^{-1}\boldsymbol{t}_{ij} = \eta^2 + \zeta^2. \tag{7}$$

In the variables (η, ζ), Eq. 3 reads

$$M_{\tilde{Z}}(t) = e^{-t\hat{d}_i\cdot\hat{d}_j}\frac{\Omega_{d-2}}{\Omega_d}\int_{\mathcal{D}} d\eta\; d\zeta\; (1-\eta^2-\zeta^2)^{\frac{d-4}{2}} e^{\frac{td}{2}(\hat{d}_i\cdot\hat{d}_j(\eta^2+\zeta^2)+\eta^2-\zeta^2)}, \tag{8}$$

with $\mathcal{D} = \{(\eta,\zeta) : \eta^2+\zeta^2 \le 1\}$. Introducing polar coordinates,

$$\eta = q\cos\phi, \qquad \zeta = q\sin\phi, \tag{9}$$

for which $d\eta\, d\zeta = q\, dq\, d\phi$, Eq. 8 becomes

$$M_{\tilde{Z}}(t) = e^{-t\hat{d}_i\cdot\hat{d}_j}\frac{\Omega_{d-2}}{\Omega_d}\int_0^1 q\; dq \int_0^{2\pi} d\phi\; (1-q^2)^{\frac{d-4}{2}} e^{td\frac{q^2}{2}(\hat{d}_i\cdot\hat{d}_j+\cos 2\phi)}, \tag{10}$$

Eq. 2 then follows upon setting $u = q^2$, so that $q\, dq = \frac{1}{2}\, du$.

(i) The function $M_{\tilde{Z}}(t, \hat{d}_i\cdot\hat{d}_j)$ in Eq. 2 is convex in the variable $\hat{d}_i\cdot\hat{d}_j$, i.e $M''_{\tilde{Z}}(t, \hat{d}_i\cdot\hat{d}_j) > 0$, $\forall t \neq 0$. Primes denote derivatives with respect to $\hat{d}_i\cdot\hat{d}_j$, single and double for first and second derivatives, respectively. Since the integrand in Eq. 62 of the main text is non-zero, it is always possible to define a probability measure

$$\pi_{\hat{d}_i\cdot\hat{d}_j}(u) = \frac{1}{Z(t,\hat{d}_i\cdot\hat{d}_j)}(1-u)^{\frac{d-4}{2}} e^{\hat{d}_i\cdot\hat{d}_j t\frac{d}{2}u} I_0\big(t\frac{d}{2}u\big), \quad u\in[0,1]. \tag{11}$$

$Z(t,\hat{d}_i\cdot\hat{d}_j)$ is the normalization function. It follows that

$$M_{\tilde{Z}}(t,\hat{d}_i\cdot\hat{d}_j) = e^{-t\hat{d}_i\cdot\hat{d}_j} C_d\; Z(t,\hat{d}_i\cdot\hat{d}_j), \tag{12}$$

with $C_d = 4\pi\frac{\Omega_{d-2}}{\Omega_d}$. It is thus obtained

$$\frac{M'_{\tilde{Z}}}{M_{\tilde{Z}}} = -t + t\frac{d}{2}\langle u\rangle_{\pi_{\hat{d}_i\cdot\hat{d}_j}}$$

$$\frac{M''_{\tilde{Z}}}{M_{\tilde{Z}}} = (-t + t\frac{d}{2}\langle u\rangle_{\pi_{\hat{d}_i\cdot\hat{d}_j}})^2 + t^2\frac{d^2}{4}(\langle u^2\rangle_{\pi_{\hat{d}_i\cdot\hat{d}_j}} - \langle u\rangle^2_{\pi_{\hat{d}_i\cdot\hat{d}_j}}) > 0. \tag{13}$$

Since $M_{\tilde{Z}}(t, \hat{d}_i \cdot \hat{d}_j) > 0$, it follows $M''_{\tilde{Z}}(t, \hat{d}_i \cdot \hat{d}_j) > 0$. For $t \neq 0$, $(\langle u^2\rangle_{\pi_{\hat{d}_i\cdot\hat{d}_j}} - \langle u\rangle^2_{\pi_{\hat{d}_i\cdot\hat{d}_j}}) > 0$ because the measure $\pi_{\hat{d}_i\cdot\hat{d}_j}$ is non-degenerate for $u \in [0, 1]$. Being $M_{\tilde{Z}}(t, \hat{d}_i\cdot\hat{d}_j)$ strictly convex, the global maximum is attained at one of the endpoints $\hat{d}_i \cdot \hat{d}_j = \pm 1$. As shown below, the maximum occurs at $\hat{d}_i \cdot \hat{d}_j = 1$, i.e. the quantity

$$\Delta = \log M_{\tilde{Z}}(t, 1) - \log M_{\tilde{Z}}(t, -1), \tag{14}$$

is positive. From Eq. 12,

$$\Delta = -2t\hat{d}_i \cdot \hat{d}_j + \log Z(t, \hat{d}_i \cdot \hat{d}_j = 1) - \log Z(t, \hat{d}_i \cdot \hat{d}_j = -1). \tag{15}$$

The function $f(\hat{d}_i \cdot \hat{d}_j) = \log Z(t, \hat{d}_i \cdot \hat{d}_j)$ is strictly convex in $\hat{d}_i \cdot \hat{d}_j$ because $f'(\hat{d}_i \cdot \hat{d}_j) = t\frac{d}{2}\langle u\rangle_{\pi_{\hat{d}_i\cdot\hat{d}_j}}$ and $f''(\hat{d}_i\cdot\hat{d}_j) = t^2\frac{d^2}{4}(\langle u^2\rangle_{\pi_{\hat{d}_i\cdot\hat{d}_j}} - \langle u\rangle^2_{\pi_{\hat{d}_i\cdot\hat{d}_j}}) > 0$. Since $f(\hat{d}_i\cdot\hat{d}_j)$ is strictly convex, it follows that $f(1) - f(-1) > 2f'(0) = t\frac{d}{2}\langle u\rangle_{\pi_0}$. The following inequality is thus established,

$$\Delta > t\frac{d}{2}\langle u\rangle_{\pi_0}. \tag{16}$$

Defining the probability distribution $\pi'_0 = \frac{1}{C_{\pi'_0}}(1-u)^{\frac{d-4}{2}}$, where $C_{\pi'_0}$ is a normalization constant, one can states $\langle u\rangle_{\pi_0} = \frac{\langle uI_0(t\frac{d}{2}u)\rangle_{\pi'_0}}{\langle I_0(t\frac{d}{2}u)\rangle_{\pi'_0}} \geq \langle u\rangle_{\pi'_0}$. The last inequality follows because u and $I_0(t\frac{d}{2}u)$ are comonotonic and hence the Chebyshev's inequality holds.[1] The average of u by the probability distribution π'_0 can be easily computed obtaining $\langle u\rangle_{\pi'_0} = \frac{2}{d}$. From Eq. 16 then it follows $\Delta > t > 0$ since t is positive by construction. For $d \geq 3$, Eq. 22 of the main text is then minimum when $\hat{d}_i \cdot \hat{d}_j = 1$.

References

(1) Agahi, H. An elementary proof of the covariance inequality for Choquet integral. *Stat. Probab. Lett.* **2015**, *106*, 173–178. https://doi.org/10.1016/j.spl.2015.07.011.
